# *N*‐Demethylsinomenine Relieves Neuropathic Pain in Male Mice Mainly via Regulating α2‐Subtype GABA_A_ Receptors

**DOI:** 10.1111/cns.70197

**Published:** 2025-01-03

**Authors:** Weiwei Rong, Xunjia Qian, Yujian Yin, Yipeng Gu, Weiyi Su, Jie‐Jia Li, Yue Xu, Hongyan Zhu, Junxu Li, Qing Zhu

**Affiliations:** ^1^ School of Pharmacy Nantong University Nantong Jiangsu China; ^2^ Provincial Key Laboratory of Inflammation and Molecular Drug Target Nantong Jiangsu China; ^3^ Affiliated Hospital 2 of Nantong University Nantong Jiangsu China; ^4^ State Key Laboratory of Quality Research in Chinese Medicine Macau University of Science and Technology Macau China

**Keywords:** analgesic effects, GABA_A_ receptor α2 subtype, *N*‐demethylsinomenine, neuropathic pain, pain‐evoked behavior, pain‐suppressed behaviors

## Abstract

**Aims:**

*N*‐Demethylsinomenine (NDSM) demonstrates good analgesic efficacy in preclinical pain models. However, how NDSM exerts analgesic actions remains unknown.

**Methods:**

We examined the analgesic effects of NDSM using both pain‐evoked and pain‐suppressed behavioral assays in two persistent pain models. Then western blot assay and immunofluorescence staining were used to investigate the effects of NDSM on the expression of the GABA_A_ receptor α2 subunit (GABRA2) and inflammatory factors in the spinal cord and brain tissues of male spared nerve injury (SNI) mice. Finally, the individual subtypes of GABA_A_Rs (α1, α2, α3, and α5) were respectively silenced by viral‐mediated knockdown to explore the involvement of subtypes of GABA_A_Rs in the effects of NDSM on the pain‐like behaviors in male SNI mice.

**Results:**

NDSM demonstrated significant analgesic effects against chronic pain both in pain‐evoked and pain‐suppressed behavioral assays. NDSM treatment significantly reversed the SNI induced down‐regulation of GABRA2 and up‐regulation of TNF‐α and IL‐1β. The analgesic effects of NDSM were completely blocked by silencing GABRA2 or partially blocked by silencing GABRA3.

**Conclusion:**

This study provided the first evidence that the analgesic effects of NDSM are mediated primarily by GABRA2 and partially by GABRA3, and the inhibition of neuroinflammation also contributes to the analgesic effects of NDSM.

## Graphical abstract

NDSM demonstrates good analgesic efficacy in preclinical pain models with fewer side effects than sinomenine. The analgesic effects of NDSM are mediated primarily by GABRA2 and partially by GABRA3. NDSM shows promise as a novel analgesic candidate for chronic pain management.

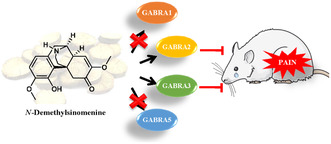

**Abbreviations**
BSA: bovine serum albumin
CFA: complete Freund' s adjuvant
DAPI: 4′; 6‐diamidino‐2‐phenylindole
GABA: Gamma‐aminobutyric acid
GABA_A_R: GABA_A_ receptor
GABRA1: α1subunit of GABA_A_R
GABRA2: α2subunit of GABA_A_R
GABRA3: α3 subunit of GABA_A_R
GABRA5: α5 subunit of GABA_A_R
GABRG2: γ2 subunit of GABA_A_R
NDSM: N‐Demethylsinomenine
NPP: neuropathic pain
NSAIDs: non‐steroidal anti‐inflammatory drugs
PFA: Polyformaldehyde solution
PMSF: phenylmethylsulfonyl fluoride
SDS: sodium dodecyl sulfate
SNI: spared nerve injury

## 1 Introduction

Chronic neuropathic pain (NPP), caused by a damage to or disease of the somatosensory system, has a very high incidence, with up to 10% of the population suffering from a variety of NPP in the world, such as peripheral NPP and central NPP [1, 2]. Currently, the pathological mechanism of NPP is recognized as maladaptive structural changes, the neuron‐immune‐glia cell interactions, and molecular signaling, which induce ectopic activity in damaged nerve and peripheral and central sensitization of nociceptive pathways leading to NPP [3, 4]. Previous reports showed that multiple factors including neuroinflammation, oxidative stress, immune cells, glial cells, ion channels, autophagy, loss of excitation‐inhibition balance, metabolic disorders, epigenetic regulation and others, contribute to the pathogenesis of NPP [4, 5]. Among these factors, neuroinflammation plays a key role in the development and maintenance of NPP. The damaged nerve will recruit and activate the immune cells to release pro‐inflammatory factors, chemokines, and other inflammatory mediators inducing inflammatory environment and peripheral nociceptive neurons sensitization, which underlies NPP. In central nerve system, glial cells activated by the aberrant signal transmission from afferent neuron produce many cells growth factors, inflammatory factors, and neuromodulators to induce neuroinflammation, which leads to central sensitization [5, 6]. NPP is regarded as one of the most difficult pain syndromes to control because of its long disease course, complex etiology and pathological mechanism, and resistance to treatment. The first‐line drugs for the treatment of NPP, such as antidepressants and antiepileptics, are only partially effective with pain reduction of about 50% for a small proportion of the patients and produce some untoward effects including dizziness, somnolence, peripheral edema, and sedation [7]. Other oral analgesics such as non‐steroidal anti‐inflammatory drugs (NSAIDs) and opioids are often chosen to relieve pain despite their limited success. The effect of NSAIDS on NPP is limited, and there are adverse reactions such as gastrointestinal injury, liver and kidney function impairment [8]. Although opioids such as morphine have good analgesic effects, their use in NPP is controversial because of their limited efficacy and their long‐term use often come with various side effects such as tolerance, dependence, addiction, and constipation [9]. There is an urgent clinical need to develop safe and effective analgesics to control pain, especially NPP.

**FIGURE 1:**
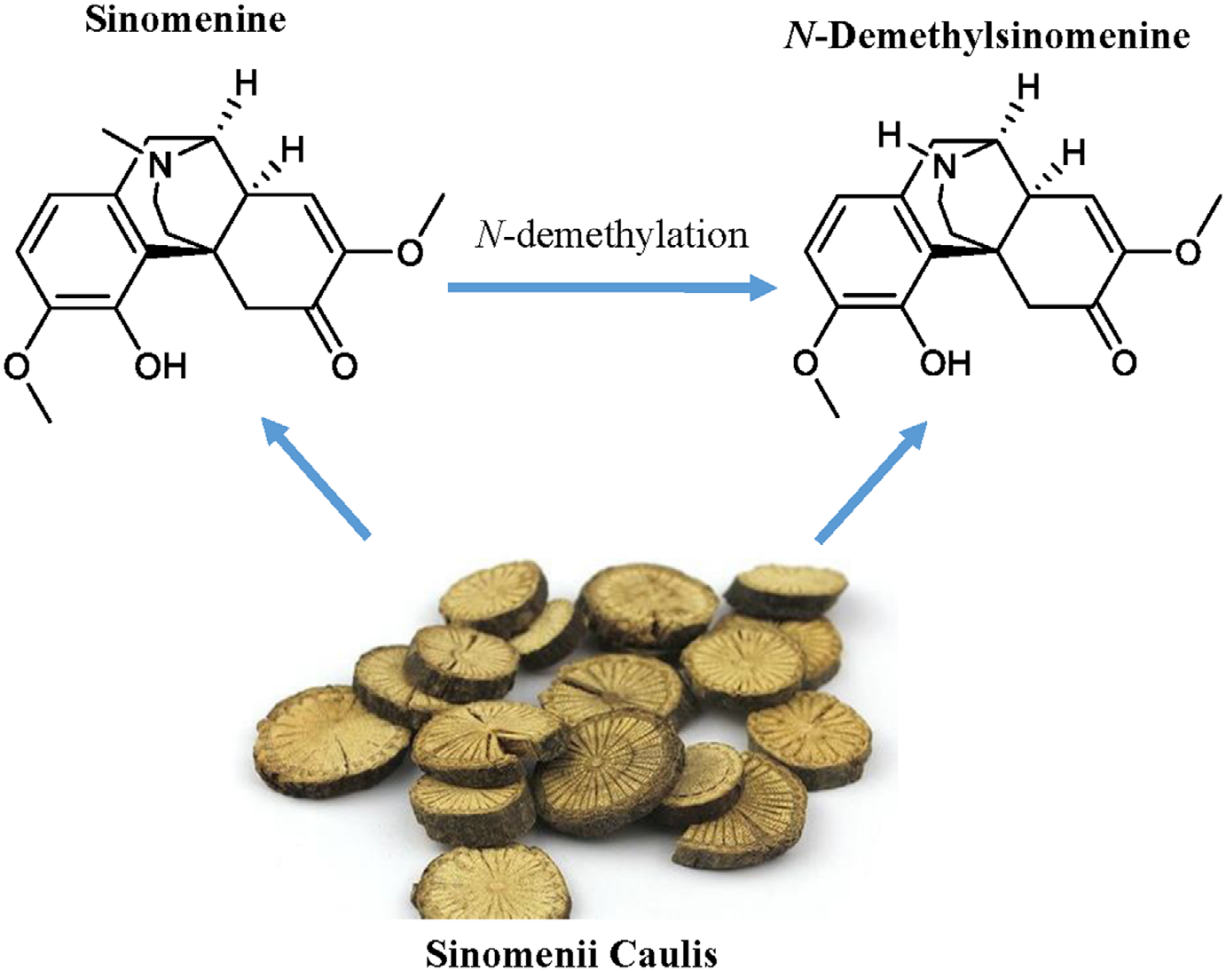
The structures of sinomenine and NDSM.

*N*‐Demethylsinomenine (NDSM) (Figure 1), the *N*‐demethylated product of sinomenine, is a natural component isolated from *Sinomenium acutum* (Thunb.) Rehd. et Wils [10]. In a mouse model of post‐operative pain [11], NDSM exerts behaviorally specific anti‐allodynia against post‐operative allodynia mediated by GABA_A_ receptors. NDSM has also shown GABA_A_ receptor‐mediated anti‐allodynic effects in mouse models of neuropathic and inflammatory pain [12]. In addition, NDSM did not show peripheral anaphylaxis like sinomenine in rats, and its sedative effect was far less than sinomenine in rats [13], suggesting that NDSM may be a more promising analgesic than sinomenine and warrants more systematic investigation.

Gamma‐aminobutyric acid (GABA) is a major inhibitory neurotransmitter, and its receptors can be classified into GABA_A_ and GABA_B_ types. Most GABA_A_ receptors (GABA_A_Rs) are heteropentameric ion channels and sensitive to benzodiazepines (BDZs). Each functional GABA_A_ receptor typically comprises of α‐, β‐, and γ or δ‐subunits in a 2:2:1 ratio. Decades of efforts have been made to decipher the subunit‐specific pharmacological actions of GABA_A_ receptors with the hope of developing subunit‐specific ligands for clinical use. For example, BDZs have a variety of clinically useful pharmacological effects. In different mice lines that carry α‐subunit‐specific point mutations, it was found that they respond differently to BDZs such as diazepam, suggesting that the pharmacological effects of BDZs such as sedation, amnesia, pain analgesia, anticonvulsant effect, and addiction potential are indeed separable and could be mediated by different GABA_A_ receptor subtypes [14, 15]. Specifically, the sedation, amnesia, anticonvulsant effect, and addiction of BDZs are primarily related to the α1 subunit of GABA_A_R (GABRA1), and the analgesic effect is mainly related to the α2 and α3 subunits (GABRA2 and GABRA3), and the α5 subunit (GABRA5) may also be involved [14]. However, the GABRA5 is also responsible for the cognitive impairment related to BDZs which is likely one of the reasons that Pfizer stopped the development of its GABA_A_ α2/α3/α5 positive allosteric modulator (PAM) Darigabat (also known as PF‐06372865) as an analgesic [16]. Ralvenius et al. [17] studied the triple GABA_A_R point‐mutated mice (express only one BDZ‐sensitive GABA_A_R subtype at a time), and found that for mechanical hyperalgesia, GABRA2 showed the strongest anti‐hyperalgesia among the subtypes. Similarly, GABRA2 also showed the strongest anti‐hyperalgesia in heat hyperalgesia and chemical nociception. Thus, GABRA2 seems to be a promising analgesia drug target. Recent developments of subtype‐selective GABA_A_ receptor PAMs raise new hope of designing better and more selective PAMs to meet clinical needs [16].

Previous studies on mice have shown that NDSM exhibits excellent anti‐allodynic effects mediated by GABA_A_Rs [11, 12]; however, the specific GABA_A_ receptor subtypes were not explored. In this study, we substantially extended our previous discoveries by examining the analgesic effects of NDSM on both pain‐stimulated and pain‐depressed behaviors and then exploring the GABA_A_ receptor subtype‐specific analgesic mechanisms of NDSM as well as its anti‐neuroinflammatory activities which may also contribute to its analgesic actions [18, 19, 20].

## 2 Material and Methods

### 2.1 Materials and Reagents

*N*‐Demethylsinomenine (NDSM, purity 98%) was purchased from Acme Bioscience Inc. (Palo Alto, CA, USA). Caffeine and complete Freund's adjuvant (CFA) were purchased from Sigma Aldrich (St. Louis, MO, USA). Ketoprofen was purchased from Aladdin Reagent Company (Shanghai, CN).

NDSM was dissolved in a mixture of 0.9% normal saline, Tween‐80, and DMSO (8:1:1); ketoprofen was dissolved in 1% Tween‐80 and caffeine was dissolved in 0.9% saline.

Antibodies used in this study were commercially obtained: IL‐1β (Rb, ab9722), GABRG2 (Rb, ab288564), GABRA2 (Rb, ab216973), and GABRA5 (Rb, ab259880) from Abcam (Cambridge, UK); GABRA1 (Rb, 12,410‐1‐AP), GABRA3 (Rb, 12,708‐1‐AP), and β‐actin (Ms, 66,009‐1‐Ig) from Proteintech Group Inc. (Chicago, IL, USA); NeuN (Ms) and TNF‐α (Rb, #11948) from Cell Signaling Technology (Danvers, MA, USA). All the secondary antibodies (Alexa Fluor 680 AffiniPure Goat Anti‐Rabbit IgG (H + L), 111–625‐144; Fluorescein (FITC) AffiniPure Goat Anti‐Rabbit IgG (H + L), 111–095‐003; Alexa Fluor 680 AffiniPure Goat Anti‐Mouse IgG (H + L), 115–625‐146; Cy3 AffiniPure Donkey Anti‐Mouse IgG (H + L), 715–165‐150) were purchased from Jackson Immuno Research (West Grove, PA, USA). For western blotting analysis, 10 μL of each primary antibody was diluted by 10 mL primary antibody diluent, while 1 μL of β‐actin and each secondary antibody was diluted by 10 mL secondary antibody diluent. For immunofluorescent staining, GABRA3, GABRA5 and IL‐1β were diluted by 50 mL primary antibody diluent, and GABRA2 and TNF‐α were diluted by 100 mL diluent, and GABRA1 was diluted by 200 mL diluent, and NeuN was diluted by 400 mL diluent.

QuickBlock Western primary/secondary antibody diluent, anti‐fluorescence quenching sealing solution, glycine, Tris (hydroxymethyl) aminomethane (Tris), sodium dodecyl sulfate (SDS), RIPA lysis buffer (Strong), phenylmethylsulfonyl fluoride (PMSF), BCA Protein Assay Kit, 4′, 6‐diamidino‐2‐phenylindole (DAPI), and bovine serum albumin (BSA) were obtained from Beyotime Biotechnology Inc. (Shanghai, CN). Polyformaldehyde solution (32% PFA) was purchased from Leagene Biotechnology Inc. (Beijing, CN). Protease and Phosphatase Inhibitor Cocktail was obtained from New Cell & Molecular Biotech Co. Ltd. (Suzhou, CN). AAV9‐Gabrg2‐RNAi (mouse, 3.79E+12 v.g/ml), AAV9‐Gabra1‐RNAi (mouse, 2.34E+13 v.g/ml), AAV9‐Gabra2‐RNAi (mouse, 2.97E+13 v.g/ml), AAV9‐Gabra3‐RNAi (mouse, 2.15E+13 v.g/ml), AAV9‐abra5‐RNAi (mouse, 1.17 E+13 v.g/ml), and CON305 (1E+13 v.g/ml) were purchased from Genechem Chemical Technology Inc. (Shanghai, CN). All of the viruses were diluted three times with 1 × PBS before injection.

### 2.2 Animals and Modeling

Male C57BL/6 mice (22 ± 2 g) were supplied by the Experimental Animal Center of Nantong University. They were raised with food and water available *ad libitum* in an air‐conditioned room with a temperature of 22°C ± 2°C, a humidity of 55% ± 10%, and a 12 h light–dark cycle for at least 7 days to adapt to the environment. The animal protocols were approved by the Institution for Animal Care and Use Committee of Nantong University and followed the United States National Institutes of Health Guide for the Care and Use of Laboratory Animals (eighth edition).

#### 2.2.1 CFA‐Induced Inflammatory Pain

The mice were randomly divided into seven groups (*n* = 8/group): the vehicle group, the CFA group, the low, medium, and high NDSM treatment groups (*i.p*., 5, 10, and 20 mg/kg/day, respectively), the caffeine (caf) treatment group (*i.p*., 10 mg/kg/day) and the ketoprofen (ket) treatment group (*i.p*., 3 mg/kg/day). The soles of both hindfeet of the mice were disinfected with alcohol, and then 20 μL CFA was injected subcutaneously into the feet. Mice in the vehicle group were injected with equal volume of normal saline. Twenty‐four hours after CFA injection, the mice's soles showed significant swelling, indicating that the model was successfully established. Thereafter, the mice in each group were injected intraperitoneally with different drug treatments daily for 7 consecutive days, while the mice in the vehicle and CFA groups were given equal volumes of normal saline with 10% Tween‐80 and 10% DMSO during this period. The daily behavioral measure was conducted 1 h after NDSM administration and 30 min after caffeine and ketoprofen administration.

#### 2.2.2 Spared Nerve Injury‐Induced Neuropathic Pain

Similarly, the mice were randomly divided into seven groups (*n* = 8/group): the sham‐operated group (sham), the spared nerve injury (SNI) group, the low, medium, and high NDSM treatment groups (*i.p*., 5, 10, and 20 mg/kg/day, respectively), the caffeine (caf) treatment group (*i.p*., 10 mg/kg/day), and the ketoprofen (ket) treatment group (*i.p*., 3 mg/kg/day).

The SNI model was established following previous literature [21] with minor modifications. Briefly, the mice were anesthetized with 2% isoflurane in oxygen at a flow rate of 3 L/min delivered via a nose cone throughout the period of surgery and an incision was made in the longitudinal direction proximal to the knee. The muscle layer was separated by blunt dissection, and the sciatic nerve branch was visible. Then a tight ligature around the tibial and common peroneal branches was made. About 1 mm of nerves below the suture was cut, and then stitch the muscle layer, and close the incision after adding a drop of lidocaine (2 mg/mL) to the wound. The sham control group received the same procedure, except that the sciatic nerve was not lapped or severed. Mice were allowed to rest for at least 48 h after the surgical procedure and treated with penicillin to prevent infection.

After SNI surgery, mice in each group were intraperitoneally injected with different drugs daily for 7 consecutive days, and the mice in the sham and SNI group were given equal volume of normal saline during this period. The daily behavioral measure was conducted 1 h after NDSM administration and 30 min after caffeine and ketoprofen administration. The mice tissues (spinal cord, cortex, and hypothalamus) were collected 1 h after NDSM administration and 30 min after caffeine and ketoprofen administration on the 7th day for western blot (WB) assay and immunofluorescence staining. These timepoints were determined based on behavioral results where peak effect was detected.

### 2.3 Behavioral Study

#### 2.3.1 Mechanical Hyperalgesia Assessment

The mice were placed individually in a cage (31.75 × 23.50 × 15.25 cm) with a floor made of wire mesh. An ascending series of von Frey filaments that delivered approximately logarithmic incremental forces (0.16, 0.40, 0.60, 1.00, 1.40, and 2.00 g) were used at a vertical angle to stimulate the plantar hind paw skin of the mouse through a wire mesh until the filaments were bent for 3 s. The mice were acclimated to the environment for at least 15 min before testing, and each filament was tested at least five times. The pain threshold assessed using the paw withdrawal threshold (PWT), that is, the lowest bending force at which the mouse retracted its paw at least three times from the filament foot retraction in five consecutive tests [22, 23].

#### 2.3.2 Burrowing Behavior

Burrowing behavior was measured as previous described [24, 25]. Briefly, as a burrowing apparatus, a standard acrylic bottle with the cap removed was placed in the mouse cage (31.75 cm × 23.50 cm × 15.25 cm), place the top of the bottle at 6 cm height, and place 50 g of corncob padding in the bottle. The mice were then put individually in the apparatus for an hour each day for 3 days to acclimatize. On the fourth day, the amount of dry padding remaining after the rat had burrowed 1 h per day was weighed and the values recorded for three consecutive days.

#### 2.3.3 Nesting Behavior

As described previously [26], six cotton balls were placed in the four corners of the mouse cage (31.75 × 23.50 × 15.25 cm) and on both sides of the center line of the long axis, that is, the cage was divided into six regions according to the location of the cotton. The mice were put individually in this environment for 3 days to acclimatize, and their nesting behavior within 60 min was recorded from day 4. If the mouse places one of the cotton balls in the area where the other cotton balls are, it is marked as 1, if the mouse moved two cotton balls out of their original area, it was marked as 2, and so on, and if all the cotton balls are collected and concentrated in one area, it is marked as 5, indicating that the nest‐building is complete.

#### 2.3.4 Wheel Running

Wheel running was measured on a polycarbonate passive running wheel (11.5 cm in diameter) with a stainless steel tube equipped with an electronic sensor that recorded the number of turns (the wheel could be turned in either direction). The mice were individually put in this environment for 3 days to acclimatize, and autonomously ran during a 12/12 h light–dark cycle. The values of their wheel running behavior were measured starting from day 4 for 1 h per day. The results were recorded continuously for 3 days [27]. The individual conducting the behavioral test was blinded to the treatment conditions.

### 2.4 Western Blotting Analysis

Total proteins from L4–L6 segments of the spinal cord, hypothalamus, and cortex of the mice were extracted with RIPA buffer. After heating the mixture in a boiling water bath for 10 min, it was centrifuged at room temperature for 10 min at 10000 r/min. The proteins in the supernatant were quantified by a BCA assay. Equal protein was loaded onto the SDS‐PAGE gel and transferred to the nitrocellulose membrane, which was then blocked with 5% fat‐free milk. The membrane was subsequently incubated with primary and secondary antibodies, after which the membrane was removed and placed in TBST solution. The ECL Western Blotting KIT was used to observe the results. Odyssey dual‐infrared laser imager was used to scan the film, select fluorescence imaging, and use ScnImage software to process and analyze the strip data.

### 2.5 Immunofluorescent Staining

Tissues from L4–L6 segments of the spinal cord, hypothalamus and cortex of the mice were collected, injected with 0.9% saline, and then re‐injected with 4% PFA and fixed for 6 h. Sucrose solution (20% and 30%) was used to gradually dehydrate the tissue, followed by frozen sections. The tissue sections were washed three times in PBS solution. A 1% Triton solution was then added and acted on for 10 min, followed by a quick wash with PBS. After drying, the sealing fluid was added and sealed at room temperature for 2 h. The sealing fluid was removed and washed three times with PBS solution. Primary antibody was then added 20 μL and incubated overnight at 4°C, and then washed with PBS solution for three times. Afterwards, the tissue sections were added to the fluorescent secondary antibody 20 μL and incubated in the dark at room temperature for 2 h. They were washed with PBS solution three times, re‐dyed with DAPI for 15 min, and washed again with PBS solution three times. The tissue section was placed on a slide in the dark, the anti‐fluorescence quencher was dropped onto the section and the slide was covered. The target proteins (green) were observed by 488 nm‐FITC channel; NeuN (pink) was observed by Cy3 channel. The images were taken with a laser confocal microscope and the data were processed and analyzed with the software Image J.

### 2.6 Gene Silencing of the GABAA Receptor Subtypes

Mice were anesthetized by intrathecal injection with a syringe between the L4 and L5 vertebral Spaces, 10 μL virus (AAV9‐Gabrg2‐RNAi, AAV9‐Gabra1‐RNAi, AAV9‐Gabra2‐RNAi, AAV9‐Gabra3‐RNAi, AAV9‐Gabra5‐RNAi, and CON305 as a negative control virus) was injected into the spine, each animal received one injection (Lu et al., 2014). One month after the virus was injected intrathecally, the 16 mice in each group were further divided into two groups: eight for SNI surgery and eight for sham surgery. Then, behavioral study was conducted 1 h after administration of NDSM (10 mg/kg). The mouse tissue was removed for WB assay and immunofluorescence staining. Virus injection gene silencing was judged to be unsuccessful if the associated protein expression in the Sham+ virus group was consistent with that in the Sham+ negative control group. If the protein expression of the Sham+ virus group was lower than that of the Sham+ negative control group, viral injection gene silencing was successful.

### 2.7 Statistical Analyses

All statistical analyses were performed using GraphPad Prism 10 software. Results were expressed as mean ± standard error of the mean (S.E.M.), and statistical evaluation was performed using the one‐way ANOVA analysis or two‐way repeated measures ANOVA analysis (Time × Treatment) followed by Bonferroni *post hoc* analysis. (*p* < 0.05 was considered statistically significant).

## 3 Results

### 3.1 Behavioral Studies

To improve the translational value of preclinical analgesic findings and reduce false positive results, both “pain‐stimulated behaviors” and “pain‐depressed behaviors which are defined in this study as behaviors such as feeding, locomotion, nesting or operant behavior” were used to evaluate analgesia effect of NDSM in this work.

#### 3.1.1 Effects of NDSM on the Behavioral Measures of Mice After CFA Injection

##### 3.1.1.1 Effect of NDSM on Mechanical Hyperalgesia in Mice

**FIGURE 2:**
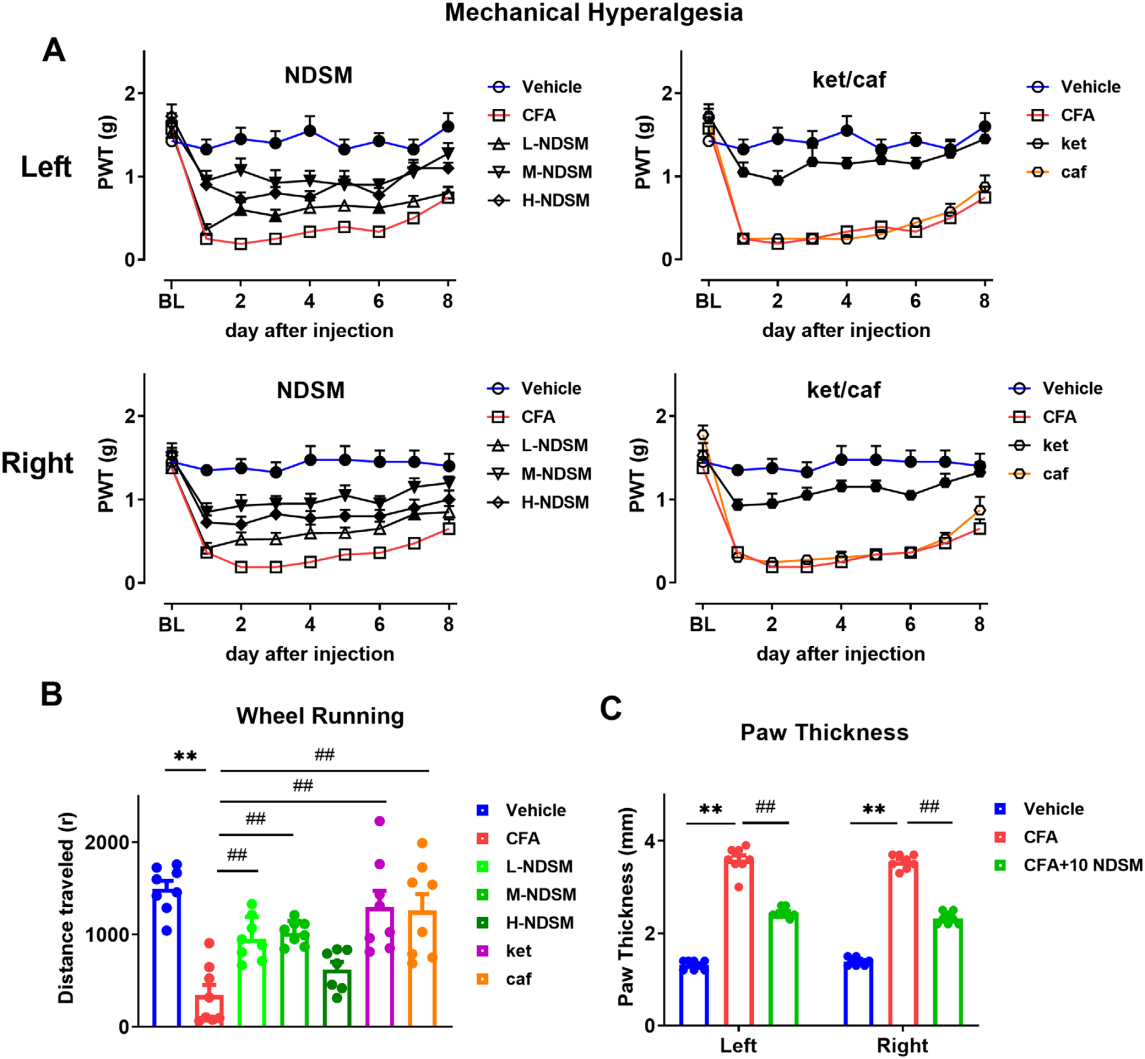
Analgesic effects of NDSM on inflammatory pain in mice induced by CFA. (A) Effect of NDSM on mechanical hyperalgesia in mice after CFA injection into the left and right feet. BL represents the baseline value before SNI surgery; (B) Effect of NDSM on wheel running in mice on the 2th day after CFA injection; (C) Effect of NDSM on paw thickness of mice on the 2th day after CFA injection. 10 NDSM: Intraperitoneal injection of NDSM 10 mg/kg/day. Statistical analysis of line graph was performed using the two‐way ANOVA analysis with repeated measures (Time × Treatment) followed by Bonferroni *post hoc* analysis (*p < 0.05 and **p < 0.01 vs. the CFA group), and statistical analysis of bar graph was performed using the one‐way ANOVA analysis followed by Bonferroni *post hoc* analysis (*p < 0.05 and **p < 0.01 vs. the Sham group; ^#^ *p* < 0.05 and ^##^ *p* < 0.01 vs. the CFA group), ANOVA *F*: Left, NDSM (row *F* (8, 315) = 29.42/column *F* (4, 315) = 97.28), ket/caf (row *F* (8, 252) = 28.91/column *F* (3, 315) = 172.7); Right, NDSM (row *F* (8, 315) = 24.44/column *F* (4, 315) = 114.6), ket/caf (row *F* (8, 252) = 28.59/column *F* (3, 315) = 195.6); wheel running, *F* (6, 48) = 11.35; paw thickness (left *F* (2, 21) = 312.2/right *F* (2, 21) = 610.2).

As shown in Figure 2A, NDSM at doses of 5, 10, and 20 mg/kg significantly increased PWT in mice injected with CFA (*p* < 0.05), with 10 mg/kg showing the most significant effect (*p* < 0.01 from day 1 to day 8 after injection). Ketoprofen (*p* < 0.05) but not caffeine could significantly increase the mechanical pain threshold in CFA mice (Figure 2A).

##### 3.1.1.2 Effect of NDSM on Wheel Running in Mice

We chose the second day post‐CFA injection to observe changes in the wheel running behavior in mice because this timepoint showed the most significant CFA‐induced decrease in this behavior. As shown in Figure 2B, the number of wheel running increased significantly after the administration of NDSM (5 and 10 mg/kg, *p* < 0.05). In this measure, ketoprofen and caffeine showed similar effects to NDSM. On the second day after CFA injection, we also measured the thickness of bilateral posterior feet of mice, and found that the CFA injection significantly increased the thickness of bilateral posterior feet in mice, which was effectively reduced by NDSM (10 mg/kg) treatment (Figure 2C).

#### 3.1.2 Effects of NDSM on the Behavioral Measures of Mice After SNI Surgery

##### 3.1.2.1 Effects of NDSM on Mechanical Hyperalgesia in SNI Mice

**FIGURE 3:**
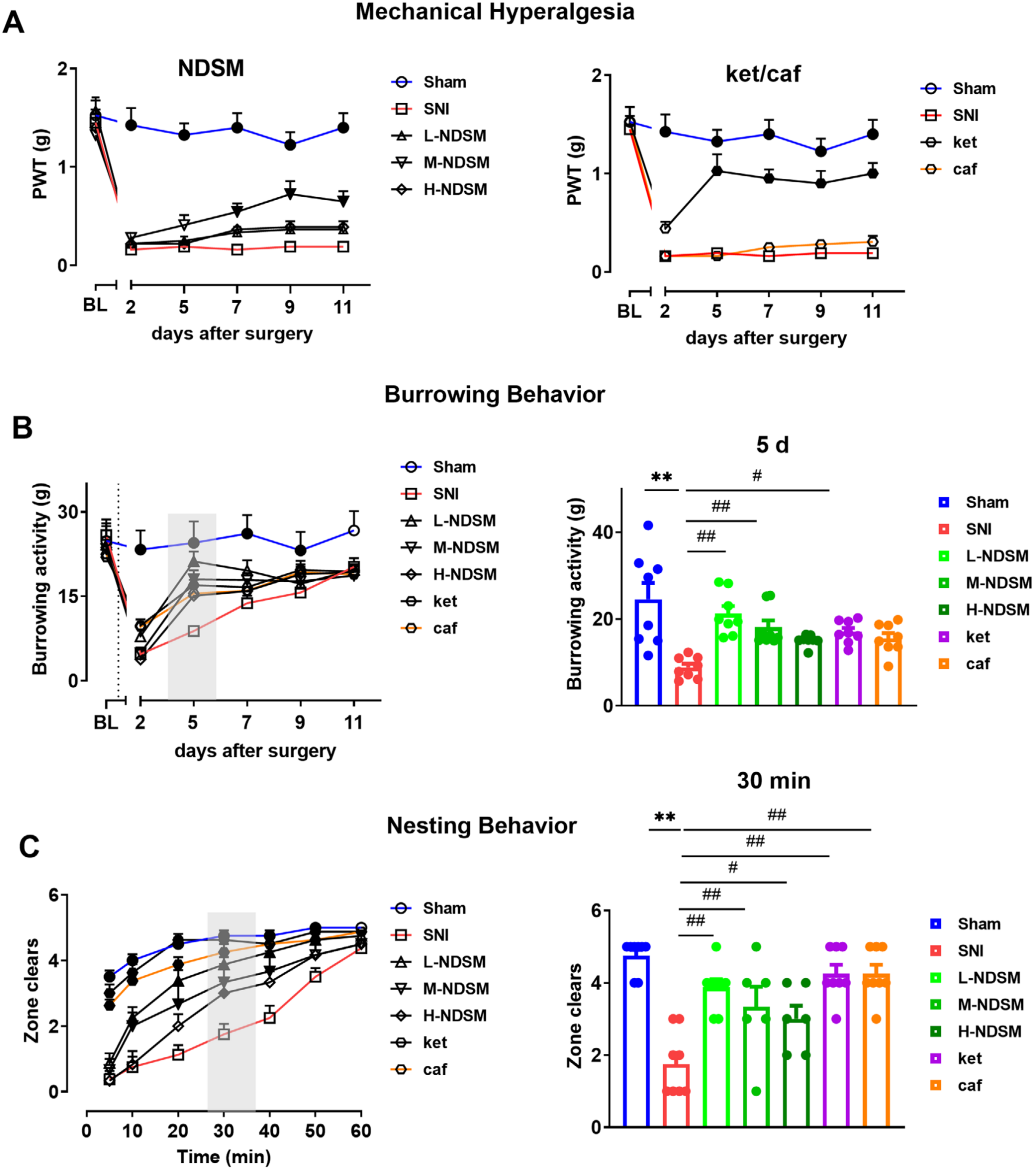
Analgesic effects of NDSM on neuropathic pain model induced by SNI in mice. (A) Effect of NDSM on mechanical hyperalgesia of the mice after SNI surgery; (B) Effect of NDSM on burrowing behavior of the mice after SNI surgery; (C) Effect of NDSM on nesting behavior of the mice after SNI surgery. BL represents the baseline value before SNI surgery; Statistical analysis of line graphs was performed using the two‐way ANOVA analysis with repeated measures (Time × Treatment) followed by Bonferroni *post hoc* analysis (*p < 0.05 and **p < 0.01 vs. the SNI group), and statistical analysis of bar graphs was performed using the one‐way ANOVA analysis followed by Bonferroni *post hoc* analysis (*p < 0.05 and **p < 0.01 vs. the Sham group; ^#^ *p* < 0.05 and ^##^ *p* < 0.01 vs. the SNI group), ANOVA *F*: NDSM (row *F* (5, 210) = 78.97/column *F* (4, 210) = 105.1), ket/caf (row *F* (5, 168) = 44.06/column *F* (3, 168) = 117.9); burrowing behavior (row *F* (5, 245) = 74.62/column *F* (6, 49) = 5.705), 5d, *F* (6, 45) = 11.88; nesting behavior (row *F* (5, 245) = 74.62/column *F* (6, 49) = 5.705), 30 min, *F* (6, 49) = 7.333.

As shown in Figure 3A, NDSM (10 mg/kg) at 7–11 days post‐SNI demonstrated the most significant improvement on the mechanical hyperalgesia threshold in mice with the PWT increasing to 0.545–0.725 g (*p* < 0.05). However, other doses of NDSM (5 and 20 mg/kg) failed to show significant effect. Ketoprofen but not caffeine significantly increased the mechanical hyperalgesia threshold on day 5 in mice. These results suggest that ketoprofen and NDSM (10 mg/kg) had significant effect on pain‐stimulated behavior in mice.

##### 3.1.2.2 Effects of NDSM on Burrowing Behavior in SNI Mice

SNI surgery suppressed burrowing behavior in mice (Figure 3B). NDSM (5 and 10 mg/kg) significantly increased SNI‐suppressed burrowing behavior in these mice. Further analysis on data from 5th day (visual inspection demonstrated the most significant effect on day 5) showed that the average burrowing weight of mice in the SNI model group was 8.825 g and NDSM (5 and 10 but not 20 mg/kg) increased the average burrowing weight to 15.09–21.21 g (*p* < 0.01). Ketoprofen also significantly alleviated the SNI‐suppressed burrowing behavior in mice (*p* < 0.05).

##### 3.1.2.3 Effects of NDSM on Nesting Behavior in SNI Mice

NDSM was able to improve the suppressed nesting behavior in SNI mice (Figure 3C). Further analysis of the nesting behavior at 30 min was conducted when the most significant effect was achieved. NDSM significantly alleviated SNI‐induced suppression of the nesting behavior in mice (*p* < 0.05). Ketoprofen and caffeine were also able to significantly reverse the suppressed nesting behavior in mice.

#### 3.1.3 Effects of Haloperidol and Caffeine on Pain‐Related Behaviors in Mice After CFA Injection and SNI Surgery

Caffeine has no known analgesic efficacy in humans yet it showed positive results in certain behavioral measures thought to be related to pain (Figures 2 and 3), suggesting that caffeine could produce false positive results in certain pain‐related behaviors. Haloperidol is a nonselective dopamine receptor antagonist that has no known analgesic efficacy in humans but also has reported false positive results in pain research that use pain‐stimulated behaviors as efficacy endpoints [28]. Thus, we tested these two compounds in both pain‐stimulated and pain‐suppressed behaviors in this study. For pain‐stimulated behaviors, caffeine (10 mg/kg) could not increase the PWT in the CFA‐treated or SNI mice (Figure S1A) whereas haloperidol (3 mg/kg) did significantly increase the PWT in CFA‐treated or SNI mice (*p* < 0.05). For pain‐suppressed behaviors in the CFA‐treated or SNI mice, haloperidol showed no significant effect but caffeine appeared to be quite effective (*p* < 0.05) (Figure S1B). Unlike haloperidol and caffeine, NDSM and the positive control drug ketoprofen showed efficacy in all the behavioral measures. Thus, the integration of both pain‐stimulated and pain‐suppressed behaviors could potentially reduce false positive discoveries and improve the translational value of preclinical findings.

#### 3.1.4 Effects of NDSM on the GABRA2 Receptor Changes in Spinal Cord and Brain Tissues of SNI Mice

##### 3.1.4.1 WB Assay

**FIGURE 4:**
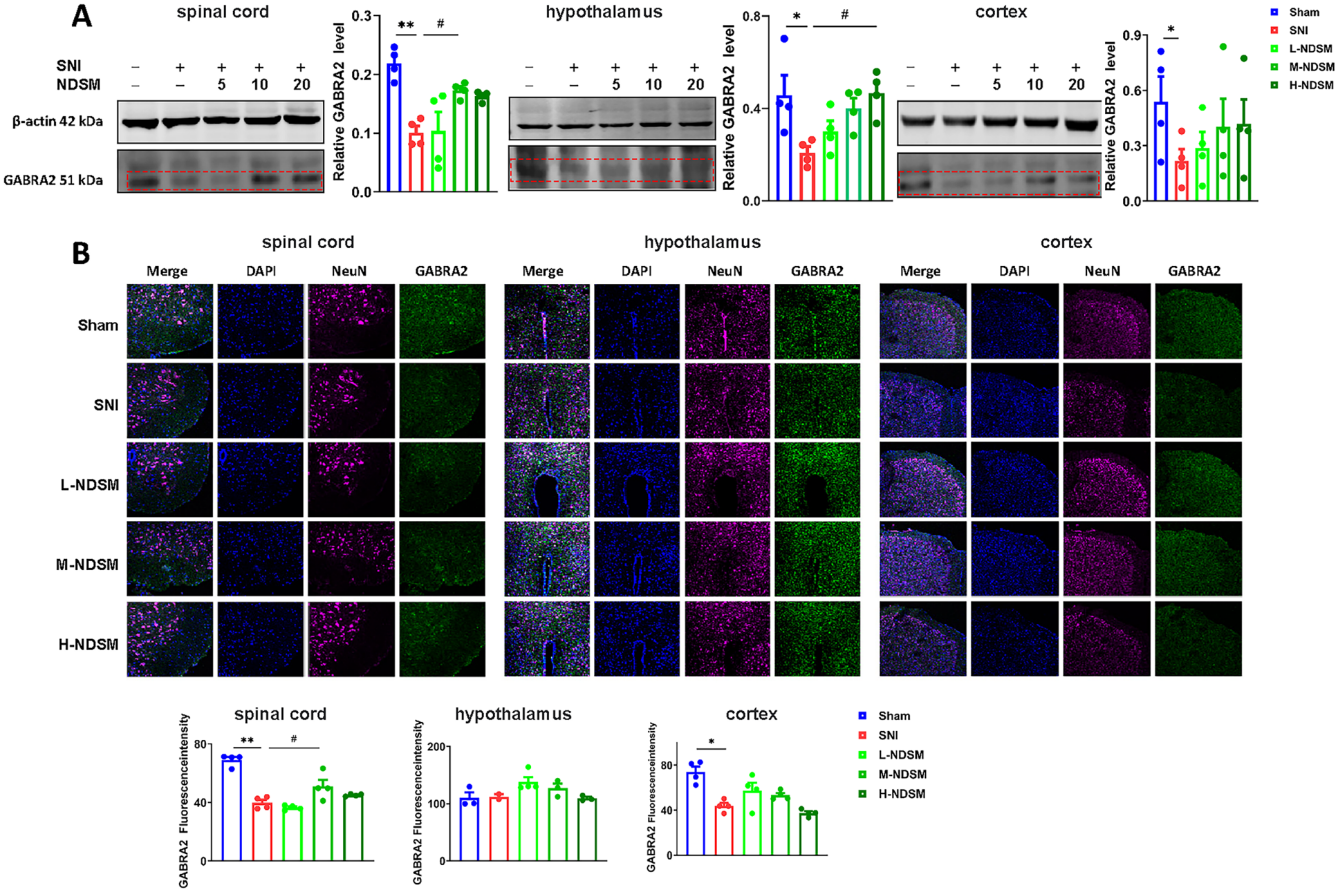
Effects of NDSM (5, 10, and 20 mg/kg/day) on GABRA2 protein expression in different tissues of SNI mice. (A) WB assay of GABRA2 expression in the spinal cord, hypothalamus and cortex of mice; (B) Immunofluorescence of GABRA2 in the spinal cord, hypothalamus, and cortex of mice. *n* = 4 (each set of data allows up to one outlier to be eliminated); Statistical analysis was performed using the one‐way ANOVA analysis followed by Bonferroni *post hoc* analysis (*p < 0.05 and **p < 0.01 vs. the Sham group; ^#^ *p* < 0.05 vs. the SNI group), ANOVA *F*: WB (spinal cord, *F* (4, 15) = 8.865, hypothalamus, *F* (4, 15) = 4.172, cortex, *F* (4, 15) = 1.067); IF (spinal cord, *F* (4, 15) = 27.37, hypothalamus, *F* (4, 15) = 2.788, cortex, *F* (4, 15) = 10.98).

The protein expression of GABRA2 in the mouse spinal cord, cortex, and hypothalamus was significantly reduced after SNI surgery, which was clearly reversed by NDSM treatment (Figure 4A). In particular, GABRA2 expression in the spinal cord was significantly increased by 10 mg/kg NDSM and by 20 mg/kg NDSM in the hypothalamus. In the cortex, the GABRA2 level was increased after the administration of NDSM; however, the upward trend did not reach statistical significance.

##### 3.1.4.2 Immunofluorescent Staining

The fluorescence of GABRA2 was significantly decreased in the spinal cord and cortex of mice after SNI surgery (*p* < 0.05) as compared with the sham group, and NDSM treatment increased the fluorescence intensity of GABRA2 with 10 mg/kg reaching statistical significance (*p* < 0.05) (Figure 4B). These results suggest that SNI surgery significantly reduced GABRA2 expression in the spinal cord, cortex and hypothalamus, and NDSM treatment reversed SNI‐induced effect.

#### 3.1.5 Effects of NDSM on the Expression of Inflammatory Factors in Spinal Cord and Brain Tissues in SNI Mice

**FIGURE 5:**
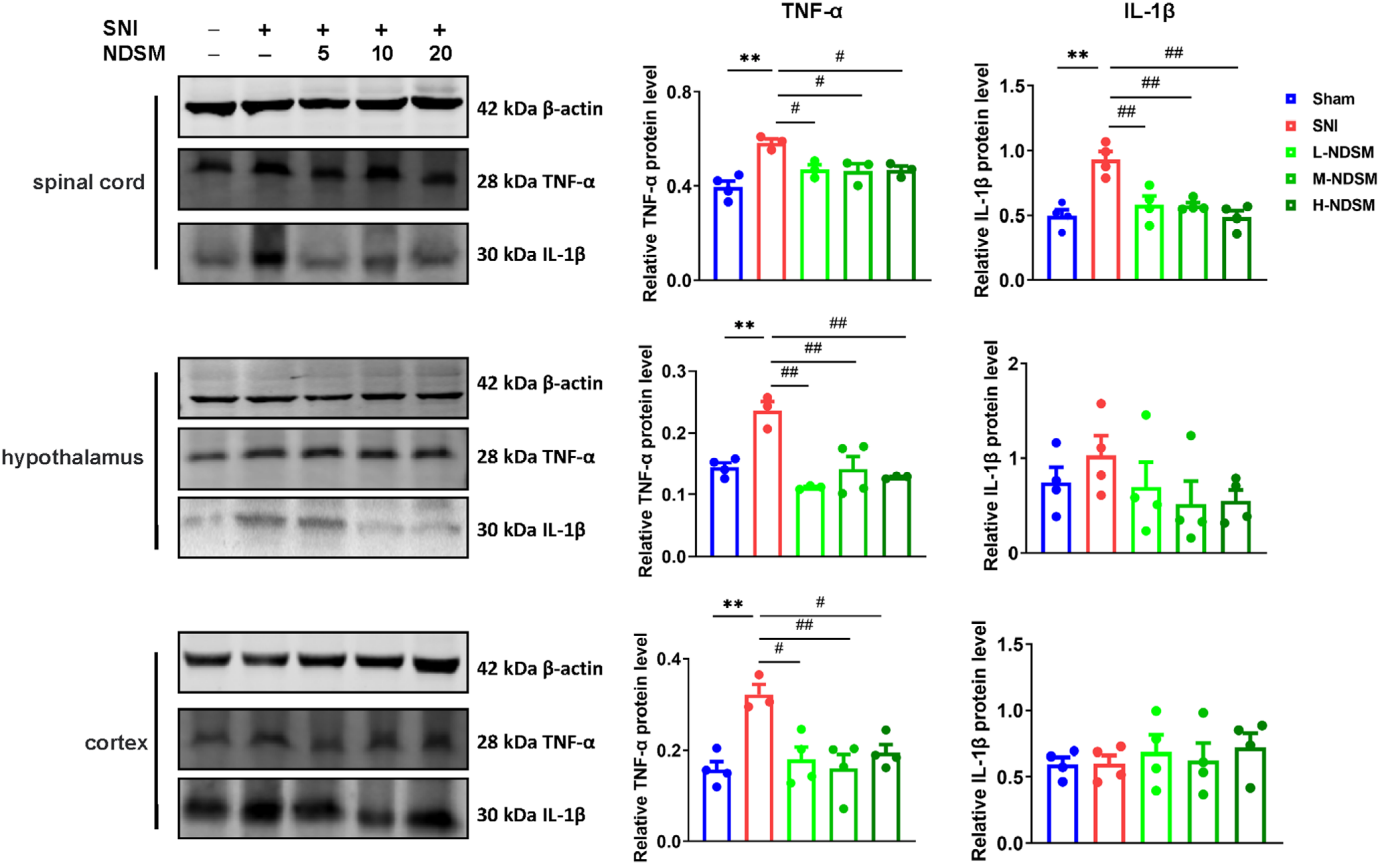
Effects of NDSM (5, 10, and 20 mg/kg/day) on TNF‐α and IL‐1β expression in different tissues of SNI mice by WB assay; *n* = 4 (each set of data allows up to one outlier to be eliminated), Statistical analysis was performed using the one‐way ANOVA analysis followed by Bonferroni *post hoc* analysis (**p < 0.01 vs. the Sham group; ^#^ *p* < 0.05 and **p < 0.01 vs. the SNI group), ANOVA *F*: Spinal cord (TNF‐α, *F* (4, 15) = 8.922, IL‐1β, (4, 15) = 12.81); hypothalamus, (TNF‐α, *F* (4, 12) = 12.13, IL‐1β, (4, 15) = 0.9764); *F* (4, 14) = 6.717, cortex, *F* (4, 15) = 0.3031.

Partial nerve injury is known to activate glial cells which leads to the increase of a variety of pro‐inflammatory factors, such as IL‐1β, IL‐6, TNF‐α, COX‐2, and iNOS [29], which plays a major role in the initiation and maintenance of SNI surgery‐induced NPP. As shown in Figure 5, NDSM (5, 10, and 20 mg/kg/day) significantly reduced the levels of TNF‐α in the mouse spinal cord, hypothalamus, and cortex as well as IL‐1β in the mouse spinal cord. These results suggest that the analgesic effects of NDSM may be partially attributable to its anti‐neuroinflammatory effects following SNI surgery.

Combining the above biochemical findings with the behavioral results, we selected 10 mg/kg NDSM as the treatment dose in the following viral‐mediated gene silencing studies.

#### 3.1.6 Gene Silencing of the GABA_A_R in the Spinal Cord and Brain Regions on the Analgesic Effects of NDSM

Previous studies [11, 12] and our results in section 3.2 have shown that NDSM exhibits significant anti‐allodynic effects mediated by GABA_A_Rs. However, the effect of NDSM on GABA_A_R‐related genes has not been explored. Thus, we next investigate whether NDSM can regulate GABA_A_Rs at the genetic level.

##### 3.1.6.1 Behavioral Study

**FIGURE 6:**
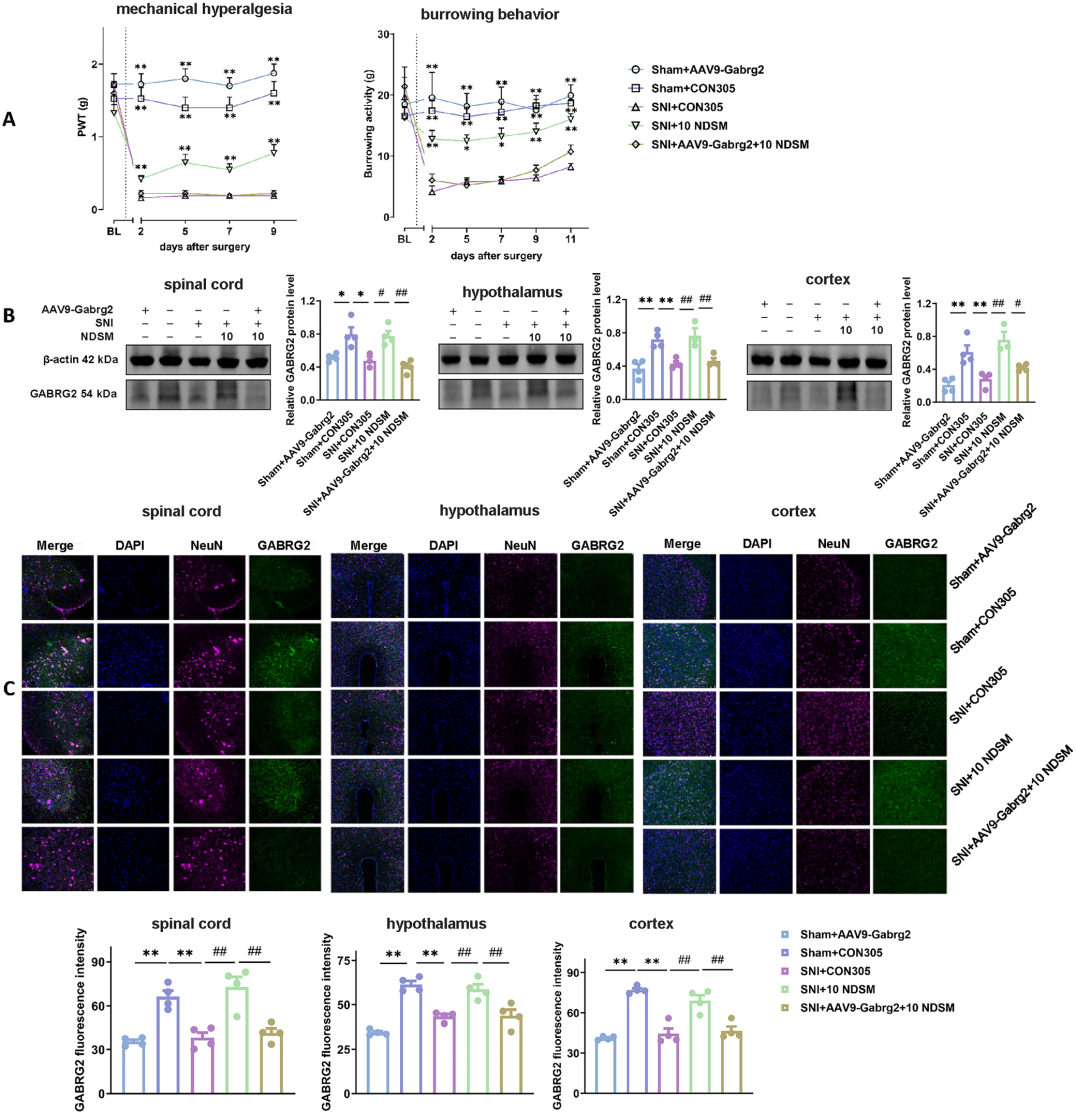
Gene silencing of GABA_A_R in the spinal cord and brain regions on the analgesic effect of NDSM. (A) Effect of intrathecal injection of AAV9‐Gabrg2‐RNAi virus on PWT and burrowing activity in mice induced by SNI surgery. BL represents the baseline value before the SNI surgery. (B): WB assay of GABRG2 expression in the spinal cord, hypothalamus and cortex of mice; (C) Immunofluorescence of GABRG2 in the spinal cord, hypothalamus, and cortex of mice; *n* = 4 (each set of data allows up to one outlier to be eliminated), 10 NDSM: Intraperitoneal injection of NDSM 10 mg/kg/day, CON305 is a negative control virus, statistical analysis of line graphs was performed using the two‐way ANOVA analysis with repeated measures (Time × Treatment) followed by Bonferroni *post hoc* analysis (*p < 0.05 and **p < 0.01 vs. the SNI + CON305 group); and statistical analysis of bar graphs was performed using the one‐way ANOVA analysis followed by Bonferroni *post hoc* analysis (*p < 0.05 and **p < 0.01 vs. the SNI + CON305 group; ^#^ *p* < 0.05 and ^##^ *p* < 0.01 vs. SNI + 10 NDSM group), ANOVA *F*: Mechanical hyperalgesia (*F* (3.206, 112.2) = 81.56, *F* (4, 35) = 51.46); burrowing behavior (*F* (5, 175) = 19.22, *F* (4, 35) = 13.63); WB (spinal cord, *F* (4, 14) = 10.70, hypothalamus, *F* (4, 14) = 12.41, cortex *F* (4, 14) = 13.46); IF (spinal cord, *F* (4, 15) = 16.75, hypothalamus, *F* (4, 14) = 24.75, cortex *F* (4, 14) = 29.64).

The baseline pain thresholds as measured by PWT were not different among mice that received intrathecal AAV9‐Gabrg2‐RNAi virus or the negative control CON305 virus. However, the PWT was significantly reduced in mice injected with CON305 following SNI surgery. The PWT of SNI mice that received NDSM (10 mg/kg) was significantly higher than that of mice injected with CON305 (*p* < 0.05). However, the effect of NDSM (10 mg/kg) was no longer evident in SNI mice with a history of receiving AAV9‐Gabrg2‐RNAi virus history (Figure 6A).

In the burrowing behavior test, similar results were found. Thus, although the pre‐SNI baseline burrowing behavior was similar among all the groups, the burrowing behavior was significantly suppressed by SNI in mice that received CON305, and 10 mg/kg NDSM was able to significantly reverse such a suppression. Importantly, the effect of 10 mg/kg NDSM was not detected in SNI mice with a history of receiving intrathecal AAV9‐Gabrg2‐RNAi virus injection (Figure 6A). This result was consistent with the above PTW result.

##### 3.1.6.2 WB and Immunofluorescence

As shown in Figure 6B,C, for the mice undergoing sham surgery, the GABRG2 levels in the spinal cord, the hypothalamus and the cortex in mice injected with AAV9‐Gabrg2‐RNAi virus were significantly lower than that in the CON305 injection group (*p* < 0.05), indicating that the AAV9‐Gabrg2‐RNAi virus significantly silenced the expression of GABRG2 in mice. SNI surgery alone also significantly reduced the GABRG2 levels in the spinal cord, hypothalamus, and cortex of the mice with a history of receiving CON305 injection and, importantly, 10 mg/kg NDSM treatment significantly increased the GABRG2 level in SNI mice. However, the GABRG2 levels in the spinal cord and brain tissues of SNI mice with a history of receiving AAV9‐Gabrg2‐RNAi virus injection were not increased even after 10 mg/kg NDSM treatment. The above results suggest that silencing GABA_A_R in the mouse spinal cord and brain can effectively block the analgesic effects of NDSM in the SNI‐induced NPP model.

#### 3.1.7 Gene Silencing of Different GABA_A_ Receptor Subtypes in the Spinal Cord and Brain Regions on the Analgesic Effects of NDSM

To further delineate which specific subtype(s) of GABA_A_R mediates NDSM‐induced analgesia, we utilized gene silencing approach to target these four different subtypes of GABA_A_R to reveal the subtype GABA_A_R mechanisms underlying NDSM‐induced analgesia in mice. The mice were divided into five groups: the first group received sham surgery and AAV9‐Gabra1/2/3/5‐RNAi virus injection, the second group received sham surgery and CON305 injection, the third group received SNI surgery and CON305 injection, the fourth group received SNI and NDSM (10 mg/kg), and the fifth group received SNI surgery, AAV9‐Gabra1/2/3/5‐RNAi virus injection and NDSM (10 mg/kg).

#### 3.1.8 Effects of Silencing the Spinal Cord and Brain GABRA1 on NDSM Analgesia

##### 3.1.8.1 Behavioral study

For mice undergoing sham surgery, a history of AAV9‐Gabra1‐RNAi virus injection did not have a significant effect on baseline PWT in mice. As expected, SNI surgery resulted in a significant reduction in PWT in the mice with or without a history of AAV9‐Gabra1‐RNAi virus injection. NDSM (10 mg/kg) significantly increased the PWT (*p* < 0.05). In the burrowing behavior test, similar results were observed (Figure S2A).

##### 3.1.8.2 WB and Immunofluorescence

As shown in Figure S2B,C, the level and fluorescence intensity of GABRA1 in both spinal cord and brain tissues of mice injected with AAV9‐Gabra1‐RNAi virus decreased more than 50% compared with the mice injected with CON305 (*p* < 0.05), suggesting the successful silencing of GABRA1. In immunofluorescence experiments, SNI surgery significantly reduced the level of GABRA1 in the spinal cord and brain tissues of mice. However, administration of NDSM (10 mg/kg) did not significantly increase GABRA1 levels in the spinal cord and brain tissue of SNI mice. Combined with the results of behavioral studies, we suggest that NDSM‐induced analgesia may not be mediated by GABRA1.

#### 3.1.9 Effects of Silencing the Spinal Cord and Brain GABRA2 on NDSM Analgesia

##### 3.1.9.1 Behavioral Study

**FIGURE 7:**
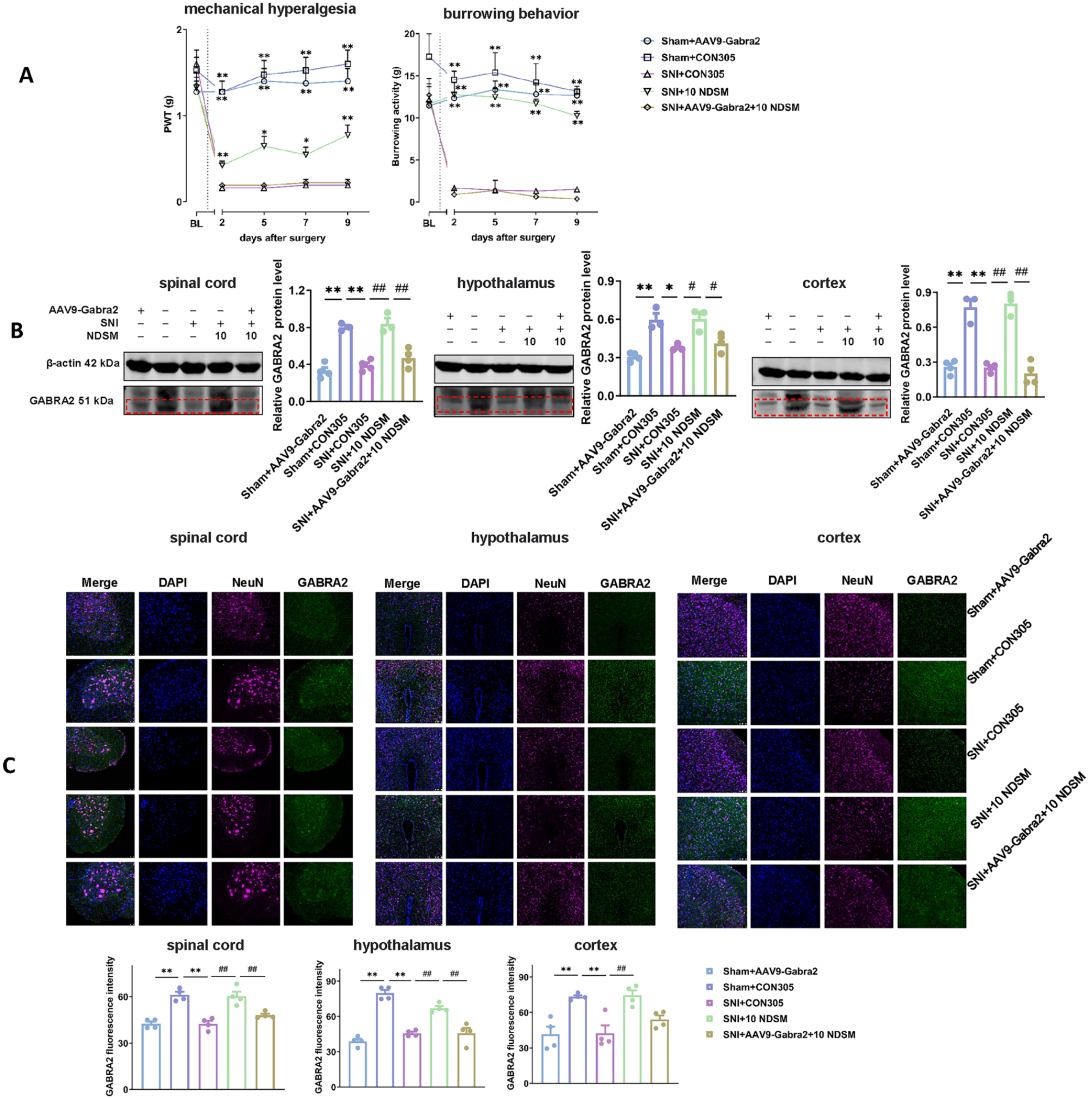
Gene silencing of GABRA2 in the spinal cord and brain regions on the analgesic effect of NDSM. (A) Effect of intrathecal injection of AAV9‐Gabra2‐RNAi virus on PWT and burrowing activity in mice induced by SNI surgery. BL represents the baseline value before the SNI surgery; (B) WB assay of GABRA2 expression in the spinal cord, hypothalamus and cortex of mice; (C) Immunofluorescence of GABRA2 in the spinal cord, hypothalamus, and cortex of mice; *n* = 4 (each set of data allows up to one outlier to be eliminated), 10 NDSM: Intraperitoneal injection of NDSM 10 mg/kg/day, CON305 is a negative control virus, statistical analysis of line graphs was performed using the two‐way ANOVA analysis with repeated measures (Time × Treatment) followed by Bonferroni *post hoc* analysis (*p < 0.05 and **p < 0.01 vs. the SNI + CON305 group); and statistical analysis of bar graphs was performed using the one‐way ANOVA analysis followed by Bonferroni *post hoc* analysis (*p < 0.05 and **p < 0.01 vs. the SNI + CON305 group; ^#^ *p* < 0.05 and ^##^ *p* < 0.01 vs. SNI + 10 NDSM group), ANOVA *F*: Mechanical hyperalgesia (*F* (2.660, 93.10) = 51.50, *F* (4, 35) = 39.09); burrowing behavior (*F* (2.346, 82.10) = 19.22, *F* (4, 35) = 57.58); WB (spinal cord, *F* (4, 13) = 29.39, hypothalamus, *F* (4, 11) = 13.85, cortex *F* (4, 13) = 42.70); IF (spinal cord, *F* (4, 15) = 22.38, hypothalamus, *F* (4, 14) = 40.78, cortex *F* (4, 14) = 10.95).

As shown in Figure 7A, there was no significant difference in the PWT of sham‐operation mice injected with AAV9‐Gabra2‐RNAi virus compared with the mice injected with CON305, while the PWT of mice undergoing SNI surgery was significantly reduced (*p* < 0.05). Interestingly, the PWT of SNI mice was significantly increased after treatment with NDSM (10 mg/kg) (*p* < 0.05), and this effect was not seen in the GABRA2 silenced SNI mice. A similar result was obtained in the burrowing behavior test in mice (Figure 7A), indicating that silencing GABRA2 in the mouse spinal cord significantly eliminated the analgesic effect of NDSM.

##### 3.1.9.2 WB and Immunofluorescence

As shown in Figure 7B, injection of AAV9‐Gabra2‐RNAi virus can significantly reduce the content of GABRA2 in the spinal cord and brain tissue of mice (*p* < 0.05), and the protein expression is about 45% of that of mice injected with CON305, indicating that the GABRA2 had indeed been silenced 1 month after the virus injection. Similarly, SNI surgery resulted in a significant decrease in GABRA2 levels in the spinal cord and brain tissue of mice, but the decreased GABRA2 levels were significantly reversed by NDSM. However, after silencing GABRA2, NDSM was unable to normalize the reduced GABRA2 level caused by SNI surgery. Similar results were also observed in immunofluorescence staining experiments (Figure 7C). Combining the results from behavioral studies, WB assay and immunofluorescence staining, we suggest that GABRA2 may be a crucial target mediating NDSM‐induced analgesia.

#### 3.1.10 Effects of Silencing the Spinal Cord and Brain GABRA3 on NDSM Analgesia

##### 3.1.10.1 Behavioral Study

**FIGURE 8:**
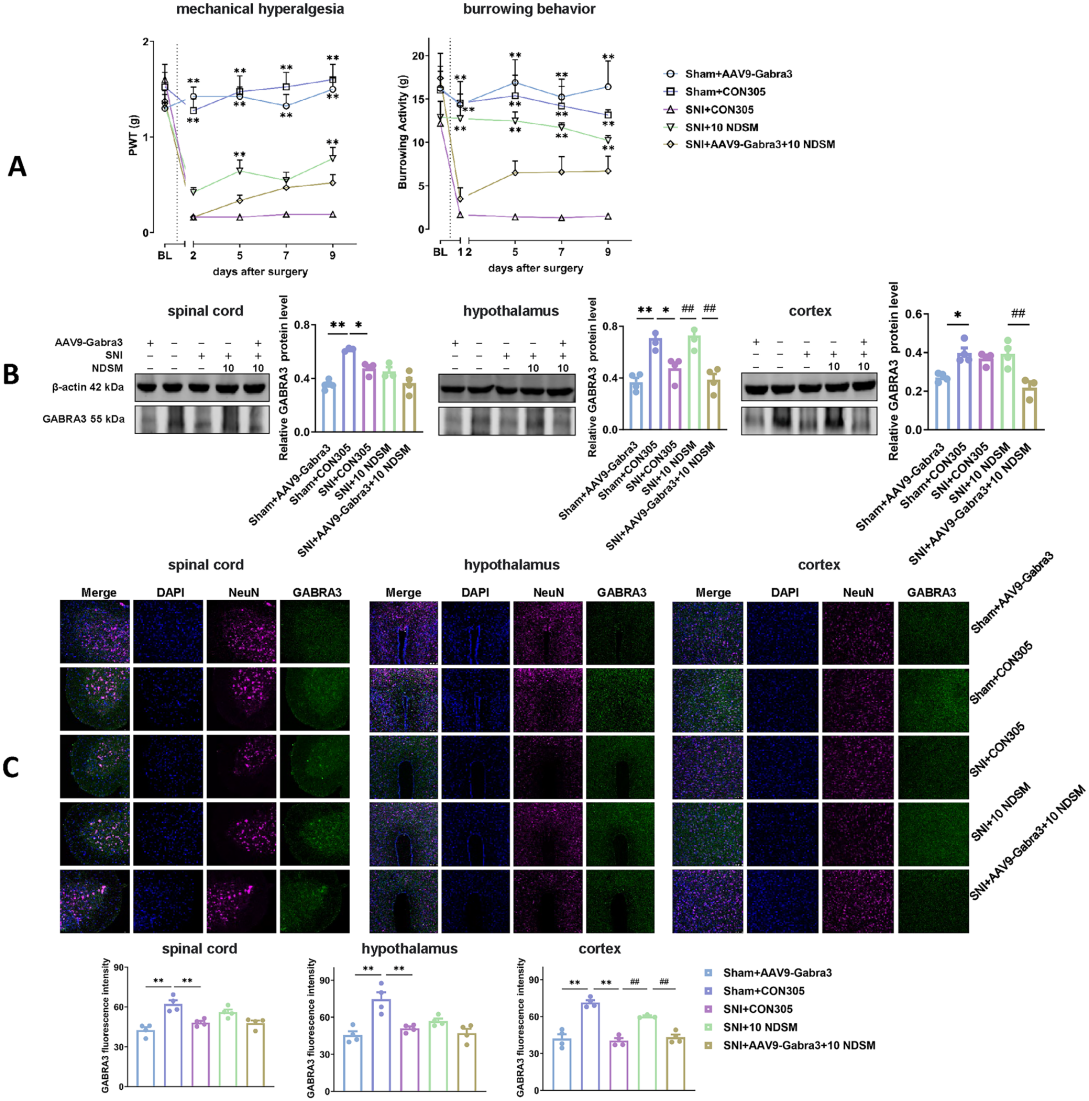
Gene silencing of GABRA3 in the spinal cord and brain regions on the analgesic effect of NDSM. (A) Effect of intrathecal injection of AAV9‐Gabra3‐RNAi virus on PWT and burrowing activity in mice induced by SNI surgery. BL represents the baseline value before the SNI surgery; (B) WB assay of GABRA3 expression in the spinal cord, hypothalamus and cortex of mice; (C) Immunofluorescence of GABRA3 in the spinal cord, hypothalamus, and cortex of mice; *n* = 4 (each set of data allows up to one outlier to be eliminated), 10 NDSM: Intraperitoneal injection of NDSM 10 mg/kg/day, CON305 is a negative control virus, statistical analysis of line graphs was performed using the two‐way ANOVA analysis with repeated measures (Time × Treatment) followed by Bonferroni *post hoc* analysis (*p < 0.05 and **p < 0.01 vs. the SNI + CON305 group); and statistical analysis of bar graphs was performed using the one‐way ANOVA analysis followed by Bonferroni *post hoc* analysis (*p < 0.05 and **p < 0.01 vs. the SNI + CON305 group; ^#^ *p* < 0.05 and ^##^ *p* < 0.01 vs. SNI + 10 NDSM group), ANOVA *F*: Mechanical hyperalgesia (*F* (4, 140) = 61.11, *F* (4, 35) = 41.12); burrowing behavior (*F* (4, 140) = 15.33, *F* (4, 35) = 13.60); WB (WB: Spinal cord, *F* (4, 13) = 14.11, hypothalamus, *F* (4, 15) = 16.32, cortex *F* (4, 14) = 12.28); IF (spinal cord, *F* (4, 15) = 12.82, hypothalamus, *F* (4, 14) = 11.47, cortex *F* (4, 14) = 33.32).

A history of AAV9‐Gabra3‐RNAi virus injection did not affect the baseline PWT in mice. However, SNI surgery significantly reduced the PWT in mice injected with CON305 (*p* < 0.05). After treatment with NDSM, PWT in the SNI mice increased to 45% of that in the sham‐operated group. However, the PWT of GABRA3 silenced SNI mice recovered slowly after NDSM treatment, and there was no significant change with the SNI group on the fifth day, but the PWT began to rise significantly after the ninth day (*p* < 0.05). In the burrowing behavior test, the results were similar. However, GABRA3 silenced SNI mice did not show a significant difference in suppressed burrowing behavior after NDSM treatment, although a slight improvement of the burrowing behavior was observed (Figure 8A). This suggests that silencing GABRA3 in the spinal cord of mice partially attenuated the analgesic effect of NDSM.

##### 3.1.10.2 WB and Immunofluorescence

Both WB and immunofluorescence results suggested that the injection of AAV9‐Gabra3‐RNAi virus into mice in the sham‐operation group could significantly inhibit the expression of GABRA3 in the spinal cord, hypothalamus and cortex of the mice (*p* < 0.05). In addition, SNI operation in mice also reduced the level of GABRA3 in relevant tissues (*p* < 0.05). Interestingly, WB and immunofluorescence results showed (Figure 8B,C) that the administration of NDSM did not recover the GABRA3 levels in the spinal cord of SNI mice, but could improve the GABRA3 levels in the hypothalamus (WB assay) and cortex (immunofluorescence staining). The discrepancies between the statistical significance results from WB and immunofluorescence can be explained by that the effect of NDSM on GABRA3 is not outstanding enough to achieve stable results of significance differences when the sample size is small (*n* = 4). Combined, we propose that GABRA3 may be involved in NDSM‐induced analgesia in the brain rather than the spinal cord.

#### 3.1.11 Effects of Silencing the Spinal Cord and Brain GABRA5 on NDSM Analgesia

##### 3.1.11.1 Behavioral Study

The results showed (Figure S3A) that the injection of AAV9‐Gabra5‐RNAi virus did not affect the baseline PWT and burrowing behavior in mice with sham‐operation, but SNI surgery significantly reduced these two behavioral indices of the mice. Administration of NDSM significantly increased the PWT and burrowing behavior in both sham (CON305) mice and in GABRA5‐silenced SNI mice.

##### 3.1.11.2 WB and Immunofluorescence

As shown in Figure S3B,C, compared with the CON305 group, GABRA5 content in both spinal cord and brain tissues of the mice injected with AAV9‐Gabra5‐RNAi virus was significantly decreased (*p* < 0.05), and the protein expression was about 50% of that in the CON305 group, indicating that GABRA5 was silenced in mice after virus injection. SNI surgery in mice significantly decreased GABRA5 in spinal cord and brain tissues. Both WB assay and immunofluorescence staining showed that administration of NDSM did not significantly increase GABRA5 levels in the spinal cord and cortex of the mice. However, NDSM can increase the GABRA5 level in the hypothalamus of SNI mice, and after silencing GABRA5, this function of NDSM disappeared. Combining results from behavioral study, WB assay, and immunofluorescence staining, we suggest that GABRA5 may not mediate NDSM‐induced analgesia.

### 3.2 Effects of NDSM on the Expression of Inflammatory Factors in the Spinal Cord of SNI Mice With Silenced GABRA2

**FIGURE 9:**
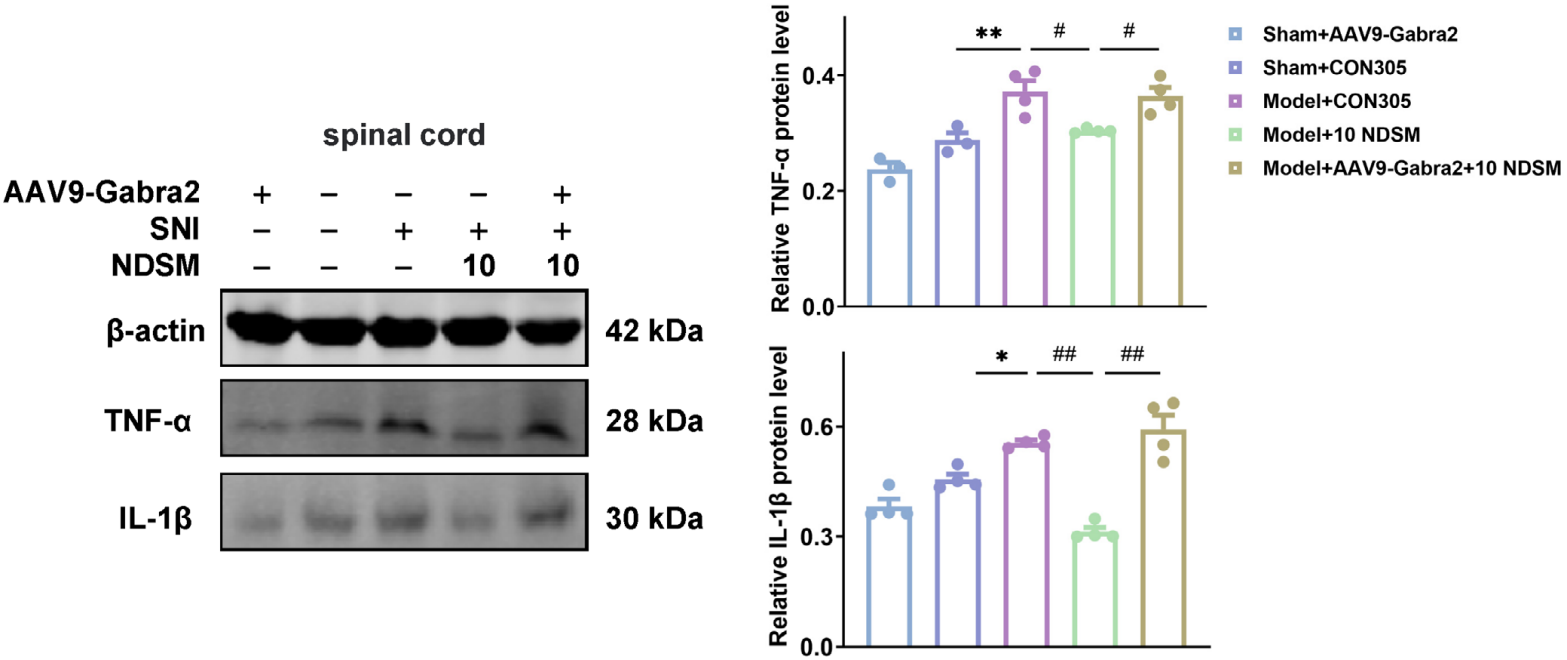
The effect of NDSM on TNF‐α and IL‐1β in the spinal cord of mice after gene silencing of GABRA2 by WB assay; *n* = 4 (each set of data allows up to one outlier to be eliminated), CON305 is a negative control virus, 10 NDSM: *Intraperitoneal injection of NDSM* 10 mg/kg/day, statistical analysis was performed using the one‐way ANOVA analysis followed by Bonferroni *post hoc* analysis (*p < 0.05 and **p < 0.01 vs. the SNI + CON305 group; ^#^ *p* < 0.05 and ^##^ *p* < 0.01 vs. SNI + 10 NDSM group), ANOVA *F*: TNF‐α, *F* (4, 13) = 16.01; IL‐1β, *F* (4, 15) = 29.34.

Because NDSM can effectively reduce the expression of inflammatory factors such as TNF‐α and IL‐1β in SNI mice, most notably in the spinal cord (Figure 5) and because silencing the expression of GABRA2 in the spinal cord blocked the analgesic effect of NDSM (Figure 9), next we examined the impact of silencing GABRA2 on pro‐inflammatory factors in the spinal cord. As shown in Figure 9, SNI surgery significantly up‐regulated the expressions of TNF‐α and IL‐1β in the spinal cord of CON305‐treated mice and NDSM treatment significantly reduced the expression levels of TNF‐α and IL‐1β in the mouse spinal cord, consistent with the previous result (Figure 4) (*p* < 0.05). Importantly, the effect of NDSM on the expression of TNF‐α and IL‐1β was not seen in the spinal cord of the GABRA2‐silenced mice (*p* < 0.05). This result suggests that the anti‐neuroinflammatory effect of NDSM was likely mediated via GABRA2 as well.

## 4 Discussion

The primary findings of this study are: (1) The false positive results may be produced in pain research that use pain‐evoked behavior (von Frey filament test) or pain‐suppressed behaviors assays (burrowing, nesting and wheel‐running behaviors) alone; (2) NDSM demonstrated true analgesic efficacy as well as ketoprofen both in pain‐evoked and pain‐suppressed behaviors in two persistent pain models (CFA and SNI models); (3) in the SNI‐induced NPP model, SNI surgery decreased the expression of GABRA2 and increased the expression of pro‐inflammatory cytokines (TNF‐α and IL‐1β), which all were reversed by NDSM treatment; (4) the analgesic effects of NDSM in SNI model were eliminated by general silencing of GABA_A_R in the mouse spinal cord or specifically silencing of GABRA2 and GABRA3 but not GABRA1 and GABRA5; (5) NDSM‐induced suppression of pro‐inflammatory cytokines in SNI mice was eliminated by spinal silencing of GABRA2. Together, these results substantially extended our previous findings by showing, for the first time, that NDSM has specific analgesic efficacy against chronic pain and the effect is primarily mediated via GABRA2 and GABRA3 subtype receptors and also involves anti‐neuroinflammatory activity. These results also support further development of NDSM as a potential analgesic drug candidate to treat pain. Because only male mice were used in this study, we caution the generalization of the findings to the female population.

One strength of the current study was the combined use of both pain‐stimulated and pain‐suppressed behaviors. This strategy has been suggested to reduce the chance of false positive results [26, 30]. Our results support this notion. In this study, we included two non‐analgesic drugs as controls to compare with the effects of NDSM. Specifically, 10 mg/kg of caffeine or 3 mg/kg of haloperidol was intraperitoneally administered to mice after CFA injection or SNI surgery, respectively, and it was found that caffeine improved pain‐suppressed behaviors but not pain‐stimulated behavior while haloperidol did the opposite. (Figures 2 and 3). Caffeine is a central nervous system stimulant [31] with no clear clinical evidence that it has analgesic effect, so caffeine is an appropriate negative control drug for analgesic studies. Given its central stimulant effect, this may explain its effect for alleviating pain‐suppressed behaviors. On the other hand, haloperidol is a classical antipsychotic drug with strong dopamine receptor antagonist activity. In certain preclinical studies, haloperidol was found to reduce certain observable pain‐like behaviors such as acetic acid‐evoked writhing; however, in preclinical assays of ICR mice, the apparent analgesic effect of haloperidol is not due to reduced sensory detection of pain, but to reduced non‐selective movement disorders in a variety of behaviors [30]. In this study, haloperidol reduced pain‐stimulated behavior but showed no significant effect in pain‐suppressed behaviors. Unlike caffeine and haloperidol, NDSM showed significant effects in both pain‐stimulated behavior and pain‐suppressed behaviors, suggesting that the observed effects are most likely due to true analgesia (pain‐reduction). These results substantially extended our previous observations that NDSM reduced pain‐stimulated behavior in CFA‐treated mice and another mouse model of NPP: chronic constriction injury model [32], and strongly suggest that NDSM could be a promising analgesic candidate that warrants further development.

GABAergic inhibition plays a vital inhibitory function in the nociceptive pathway in the spinal cord or brain, including NPP [33]. GABA_A_ receptors are anion‐selective channels. Binding of GABA to this receptor family will open the intrinsic chloride channel that induces membrane hyperpolarization to reduce neuronal excitability [34]. GABA_A_ receptors are mainly located in neuron of spinal cord and brain. GABA_A_ receptors containing the α1–3 subunits localize to postsynaptic sites, whereas GABA_A_ receptors containing the α4–6 subunits can localize to extra synaptic sites [35]. Previous reports showed that spinal injection of GABA_A_ receptors agonists relieves pain in various rodent models of inflammatory and NPP [36]. In our previous work, we had limited evidence suggesting that GABA_A_ receptor may mediate the analgesic actions of NDSM as its effect was blocked by the non‐selective GABA_A_ receptor antagonist bicuculline. However, given the complexity of GABA_A_ receptors and their involvement in pain processing [37], it is crucial to understand the specific subtypes of GABA_A_ that mediate NDSM‐induced analgesia. One challenge for solving this puzzle is that there is currently no subtype‐specific GABA_A_ receptor antagonist available. Toward this end, we employed gene silencing technique by specifying targeting different GABA_A_ receptor subtypes. WB assay and immunofluorescence staining were used to confirm the silencing efficiency and correlate with behavioral results.

Positive allosteric modulators at the BDZ‐binding site of GABA_A_ receptors (GABA_A_R) enhance synaptic inhibition through four subtypes (α_1_, α_2_, α_3_, and α_5_) of GABA_A_R. Our results showed that the silencing of GABRA1 and GABRA5 did not inhibit the analgesic effects of NDSM both in pain‐stimulated and pain‐suppressed behaviors, despite biochemical evidence that the respective receptor subtypes were efficiently silenced by roughly 50%. In contrast, silencing GABRA2 and GABRA3 can fully or partially block the analgesic effects of NDSM. These results suggest that GABRA2 or GABRA3 are the primary mediators of NDSM‐induced analgesia, which may represent novel analgesic drug targets, as recent review summarized [37]. It should be noted that current results did not provide direct evidence that NDSM is a GABA_A_ receptor orthostatic agonist or a PAM. A direct confirmation of such a mechanism requires in vitro receptor binding and electrophysiological verification using GABA_A_ receptor‐expressing cells. Nonetheless, our results provide proxy evidence that NDSM exerts antinociception predominantly via GABRA2 and partially via GABRA3. Current evidence indicated that GABRA2 partially mediates the anxiolytic‐like action, while GABRA2 together with GABRA3 mediate antinociceptive and muscle relaxant actions. However, GABRA3 seems to not mediate anxiolytic‐like effect [38]. NDSM also improved the pain‐depressed behavior in SNI model, which seems to be related the antidepressive‐like or anxiolytic‐like action. This may contribute to the reason for the differences in the effects of NDSM between GABRA2 and GABRA3.

Inflammation after neuropathy induces macrophages to activate and migrate to nerves and dorsal root ganglia, causing pain hypersensitivity through the release of pro‐inflammatory cytokines (e.g., TNF‐α). After peripheral and central neuropathy, NPP is also caused by some immunomodulators released by activated microglia in the central nervous system [39]. For instance, the initiation and maintenance of NPP in animal models involves neuro excitatory signals including IL‐1β, TNF‐α and IL‐6 [40]. Antibodies against cytokines have antinociceptive efficacy in preclinical studies [41]. Considering the effect of inflammatory factors on NPP, we examined the expression of TNF‐α and IL‐1β in SNI mice and the effect of NDSM. It was found that the expression of TNF‐α and IL‐1β was significantly increased by SNI surgery and this increase was significantly blunted after NDSM treatment, suggesting that NDSM‐induced anti‐neuroinflammatory action may also contribute to its analgesic efficacy in the SNI mice. In consistent with this, we found that silencing GABRA2 completely blocked the effects of NDSM in suppressing neuroinflammatory cytokines in SNI mice. Pro‐inflammatory factors such as TNF‐α and IL‐1β are well‐characterized pain sensitizers that contribute to various chronic pain conditions. Currently, there is little document reporting the relationship between the GABRA2 and the neuroinflammation. Only limited investigations showed that GABA_A_ receptor agonists are known to inhibit while GABA_A_ antagonists increase microglia activation and the release of pro‐inflammatory cytokines such as TNF‐α [42, 43, 44]. Glial cells including microglia and astrocyte can be activated by the aberrant signal transmission from afferent neuron to induce neuroinflammation [6]. Thus, it is speculated that NDMS directly or indirectly activates selective GABA_A_ receptors (primarily α2‐subunit but also α3‐subunit‐GABA_A_ receptors) to reduce the neuronal excitability thereby suppressing activated microglia and subsequently its release of cytokines.

The spinal cord, hypothalamus, and cortex are the key sections of nociceptive pathway. The afferent inputs from the periphery nerve were transmitted along nociceptive pathways from the spinal cord to the brain. The hypothalamus plays as a key node to transmit the signal from spinal cord to cortex in some important nociceptive pathways such as spinothalamic tract pathways and spinoreticulothalamic pathways. The alterations in spinal dorsal horn and supraspinal sites (such as hypothalamus and cortex) are involved in the central sensitization leading to NPP [4]. Our results showed that NDSM can increase the GABRA2 expression in spinal cord, hypothalamus, and cortex, but only recover the GABRA3 expression in hypothalamus and cortex not in spinal cord (Figures 7 and 8). Previous report indicates a selective GABRA2 modulator exerts antihyperalgesia exclusively through spinal action and does not involve supraspinal sites. However, it cannot be excluded whether GABRA3 also contribute through a supraspinal site [36]. Thus, we speculated spinal GABRA2 and supraspinal GABRA3 may exert synergistic effect that contributes to the antinociception of NDSM against NPP, which warrants further investigations.

## 5 Conclusions

This study extended our previous studies by demonstrating that the incorporation of pain‐depressed behaviors with pain‐evoked behaviors can to the maximum extent reduce the false positive results and NDSM exhibits true significant analgesic effect in preclinical pain models. This study also provided the first mechanistic evidence that NDSM‐induced analgesia was primarily mediated by GABRA2 and partially by GABRA3 as long as its anti‐neuroinflammatory effects. Taken together, our series of work strongly suggest that NDSM is a promising analgesic drug candidate and warrants further study.

## Author Contributions

Q.Z. designed research; W.R., X.Q., Y.Y., Y.G., W.S., and J.L. performed research; Y.X. contributed new reagents; H.Z. contributed analytic tools; W.R. and X.Q. analyzed data; W.R. wrote the draft; Q.Z. and J.L reviewed and edited the manuscript; Q.Z. and J.L managed the project and acquired the funding. All authors have read and agreed to the published version of the manuscript.

## Conflicts of Interest

The authors declare no conflicts of interest.

## Supporting information


Data S1.

## Data Availability

The data that support the findings of this study are available on request from the corresponding author upon reasonable request.
