# Supplementary material for: N‐Demethylsinomenine Relieves Neuropathic Pain in Male Mice Mainly via Regulating α2‐Subtype GABAA Receptors
Source: CNS Neurosci Ther. 2025 Jan 3;31(1):e70197. doi: 10.1111/cns.70197 (PMC11696256; doi:10.1111/cns.70197)
Supplement: Supplementary file 1 — Data S1. [file CNS-31-e70197-s001.zip › Supplemental Files of original WB.pdf]

Figure 4

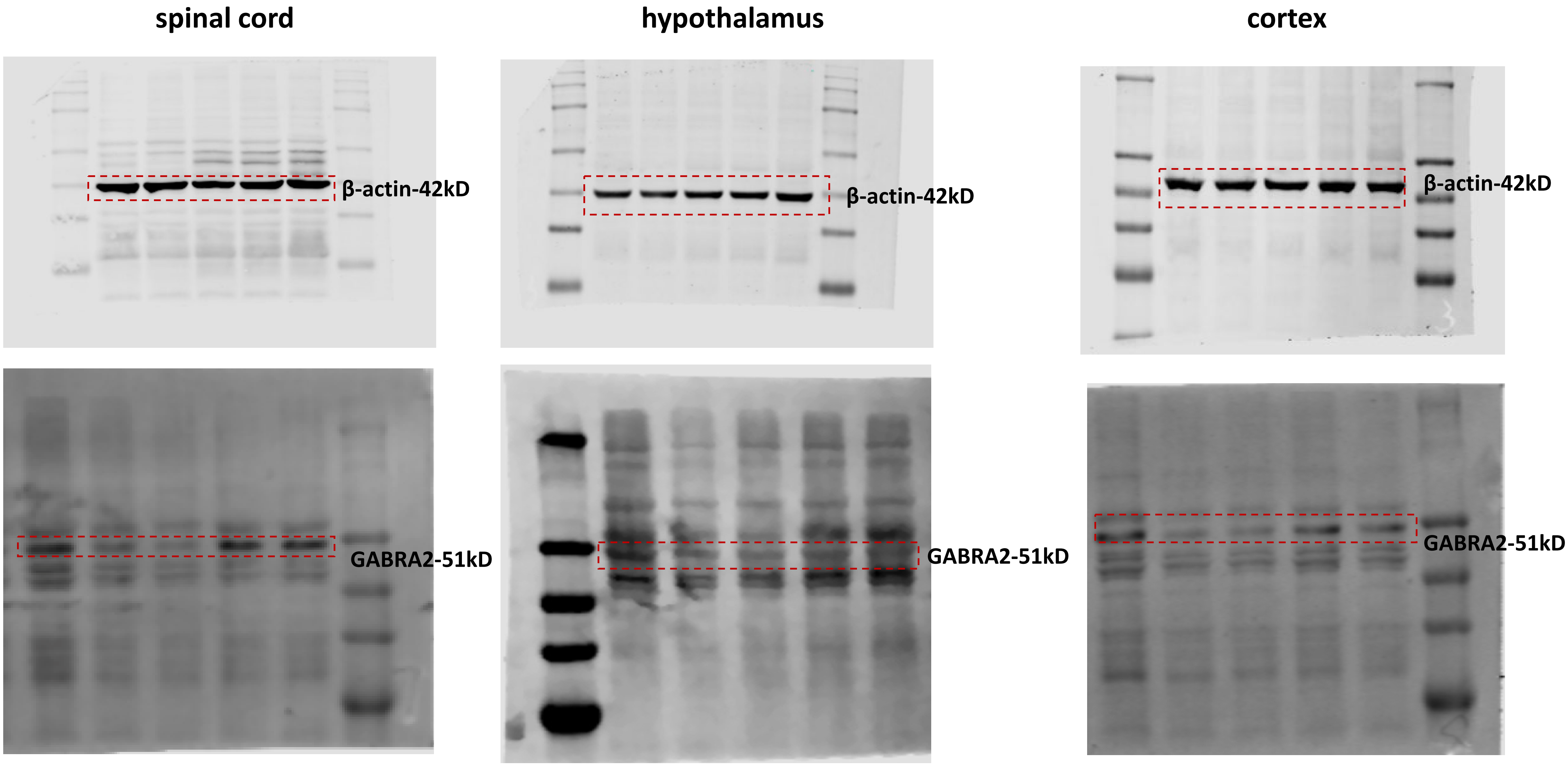
spinal cord
hypothalamus
cortex
β-actin-42kD
β-actin-42kD
β-actin-42kD
GABRA2-51kD
GABRA2-51kD
GABRA2-51kD

Figure 5

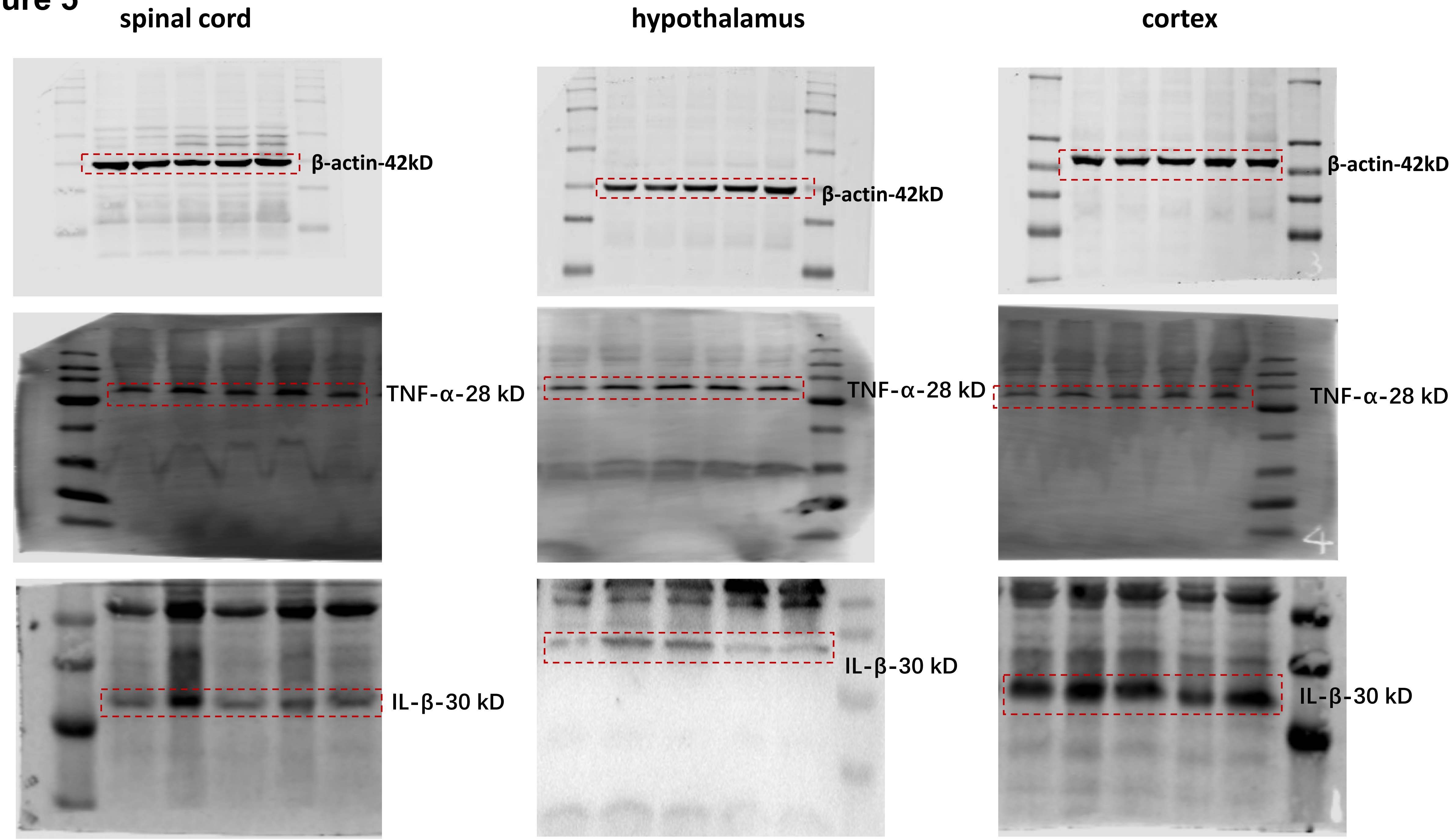

spinal cord
hypothalamus
cortex
β-actin-42kD
β-actin-42kD
β-actin-42kD
TNF-α-28 kD
TNF-α-28 kD
TNF-α-28 kD
IL-β-30 kD
IL-β-30 kD
IL-β-30 kD

Figure 6

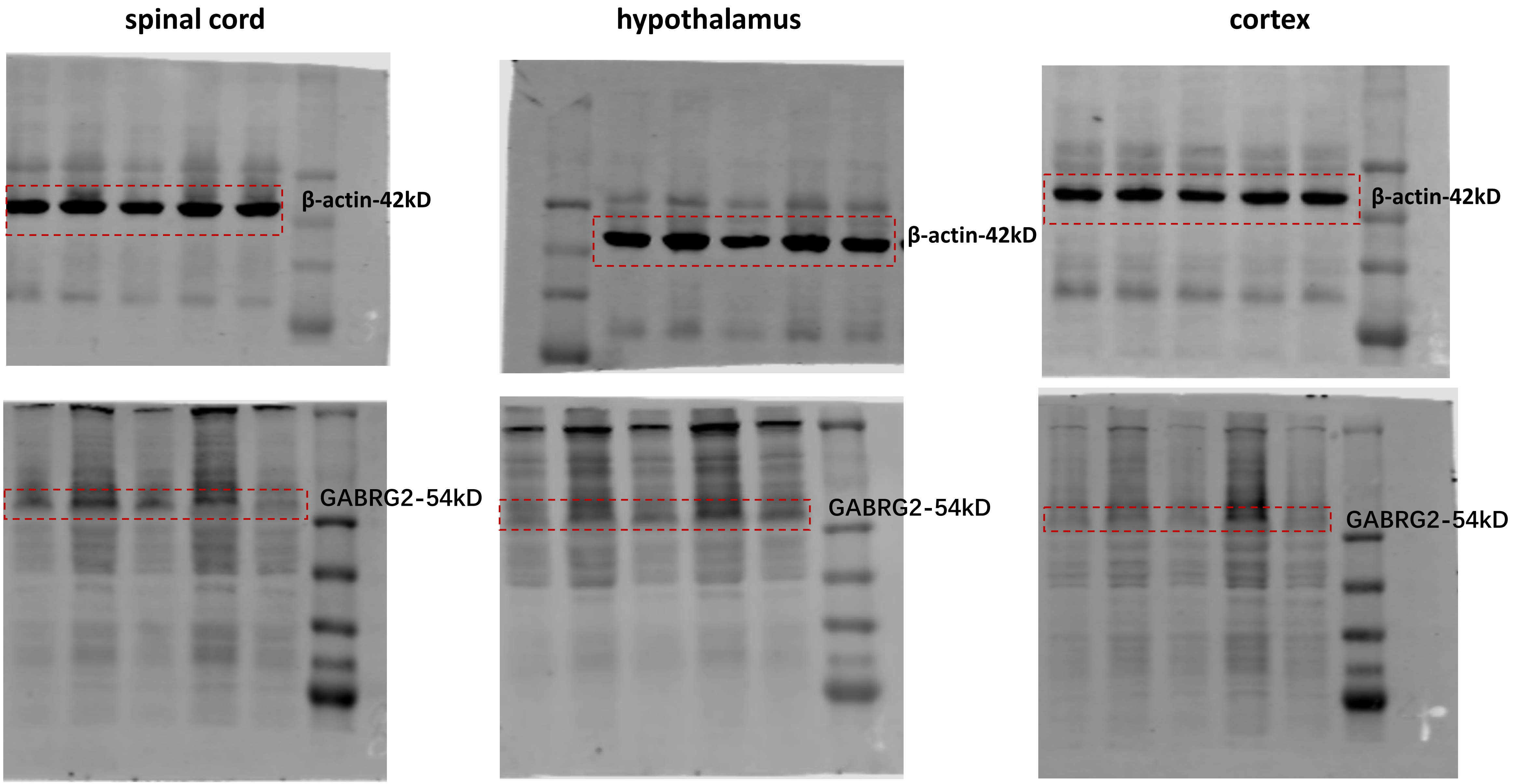
spinal cord
hypothalamus
cortex
β-actin-42kD
β-actin-42kD
β-actin-42kD
GABRG2-54kD
GABRG2-54kD
GABRG2-54kD

Figure 7

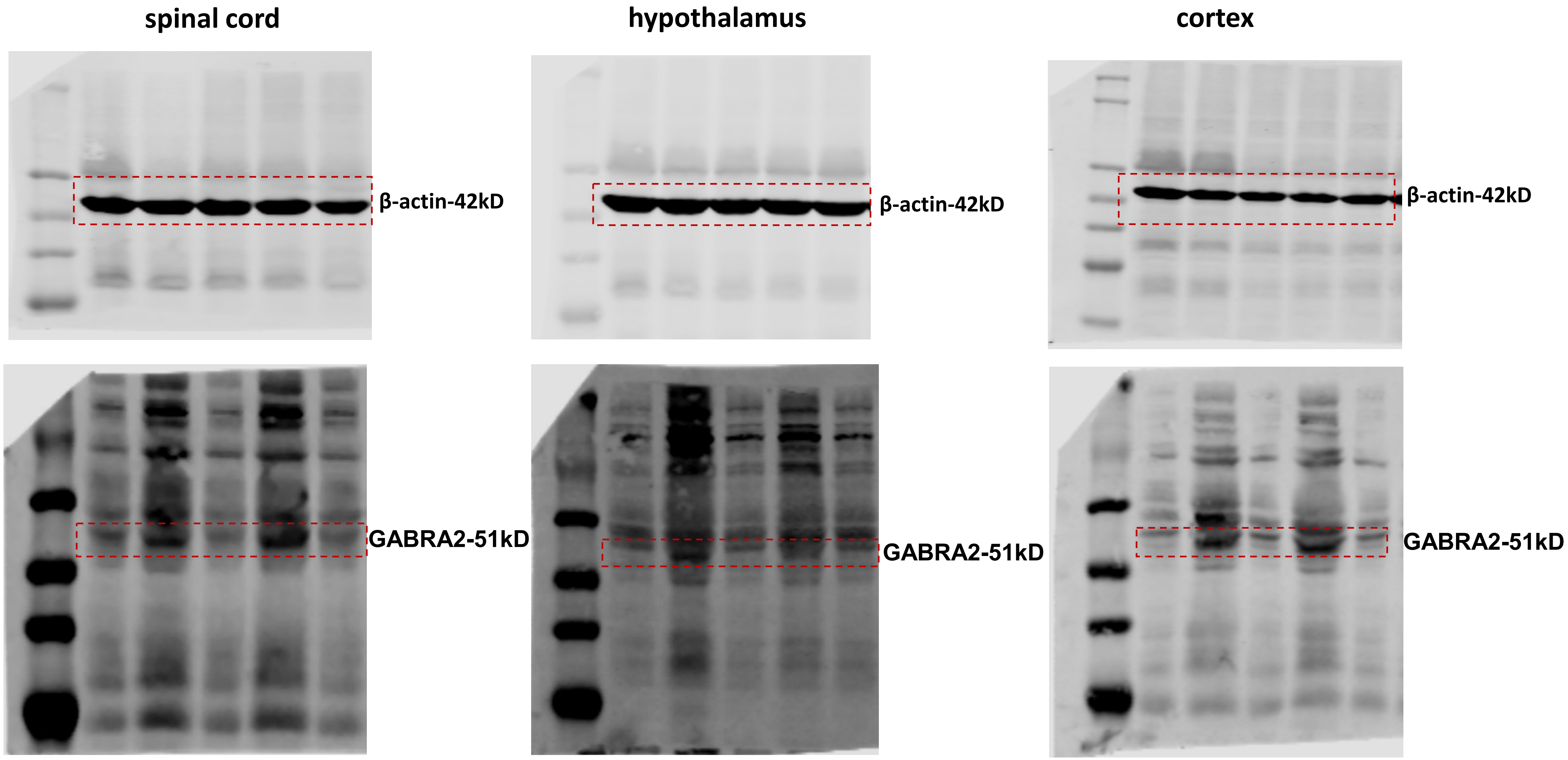
spinal cord
hypothalamus
cortex
β-actin-42kD
β-actin-42kD
β-actin-42kD
GABRA2-51kD
GABRA2-51kD
GABRA2-51kD

Figure 8

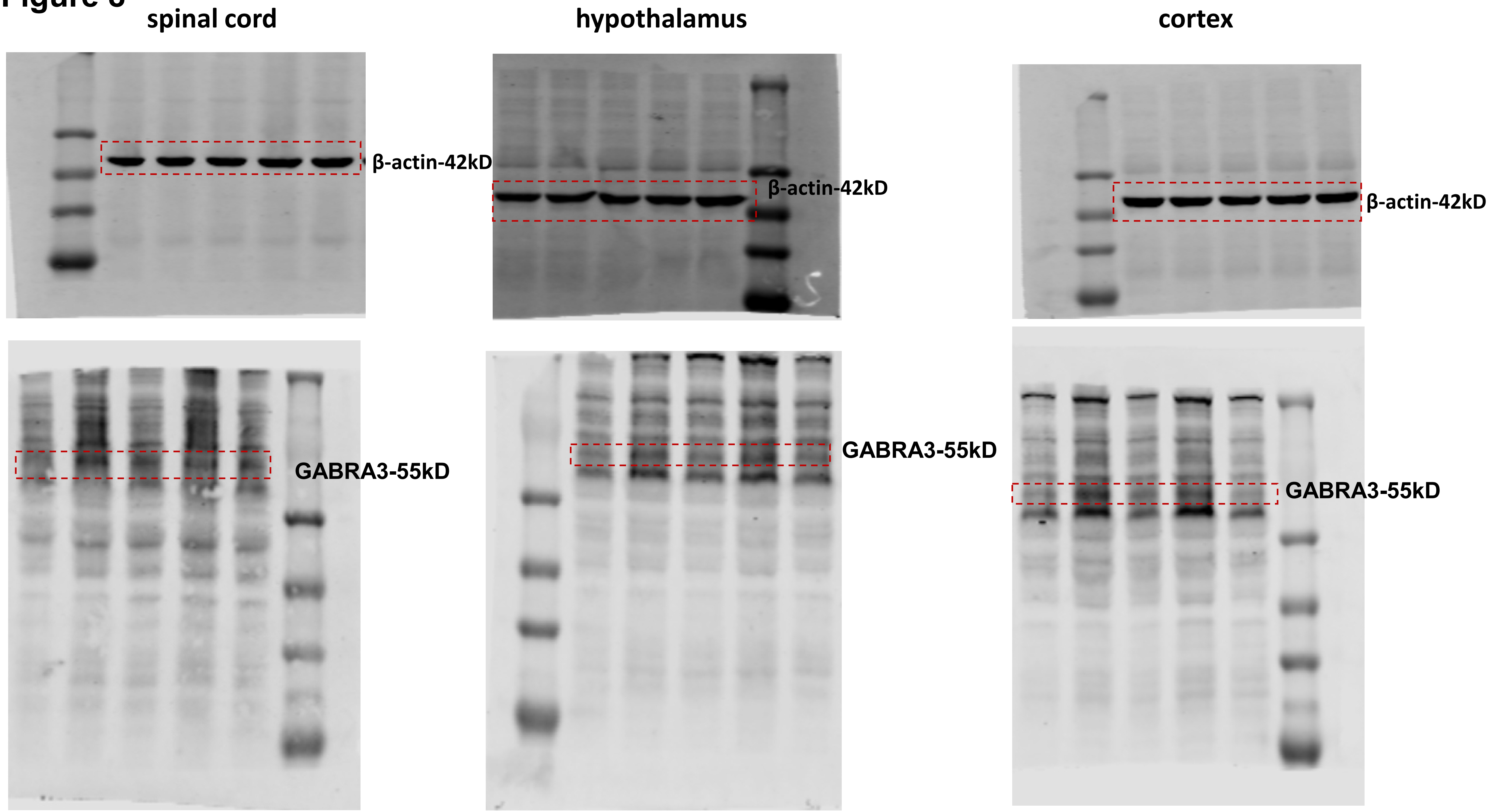
spinal cord
hypothalamus
cortex
β-actin-42kD
β-actin-42kD
β-actin-42kD
GABRA3-55kD
GABRA3-55kD
GABRA3-55kD

Figure 9

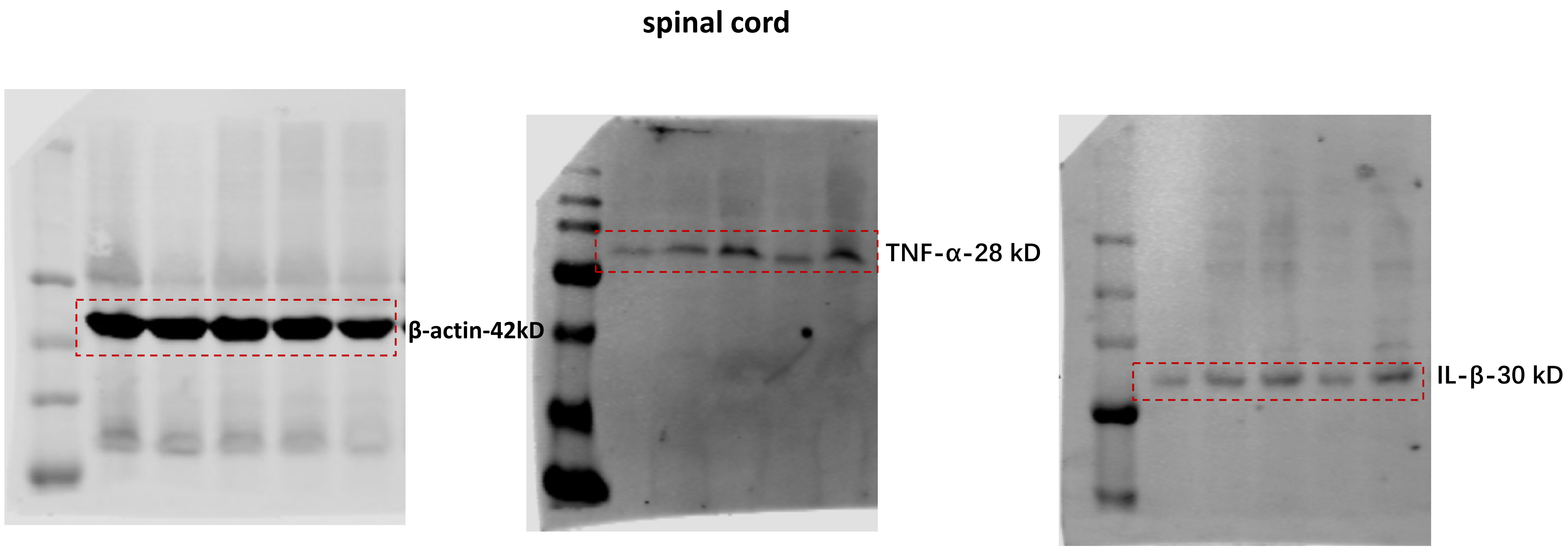

Figure S2

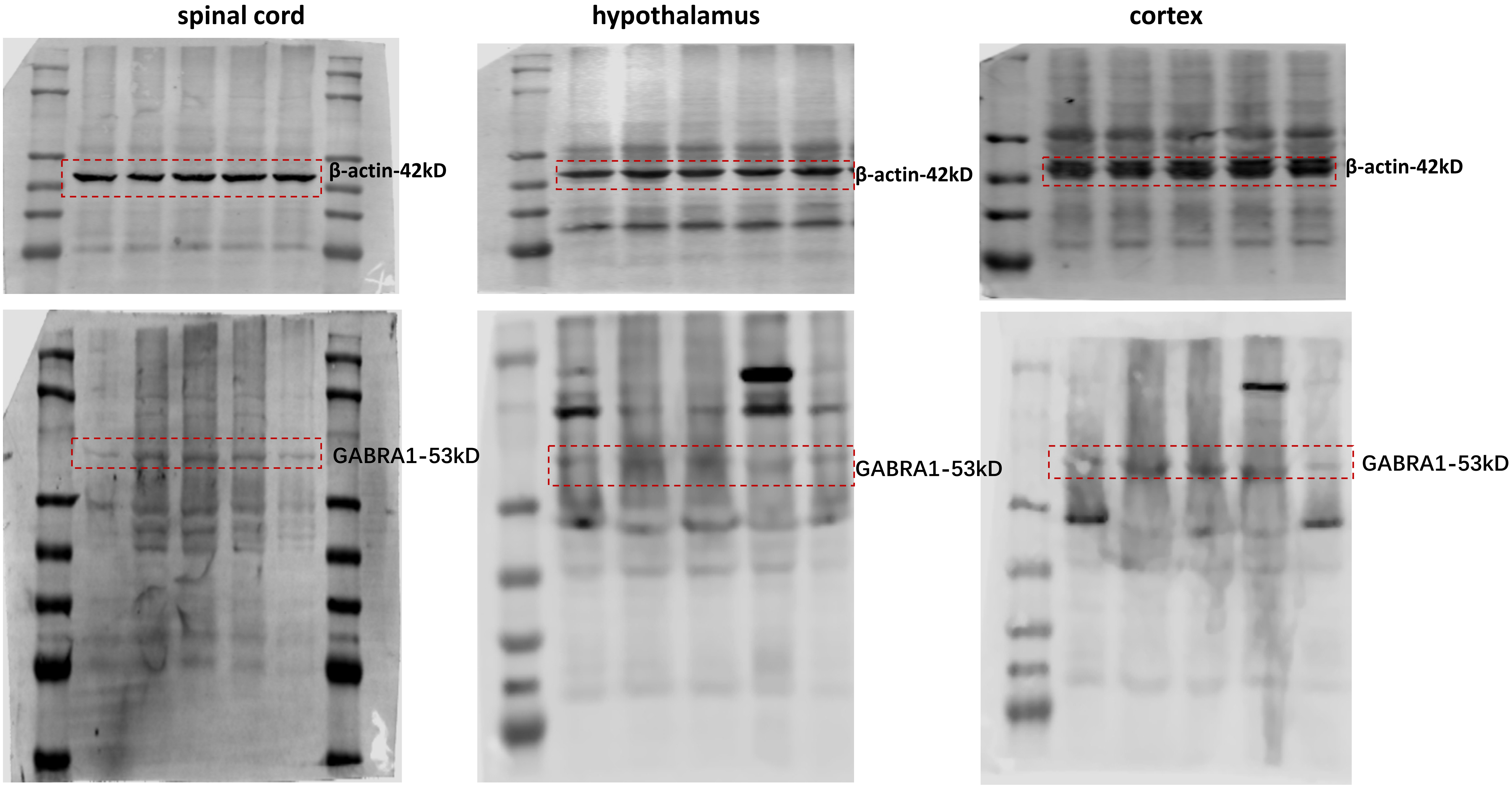
spinal cord
hypothalamus
cortex
β-actin-42kD
β-actin-42kD
β-actin-42kD
GABRA1-53kD
GABRA1-53kD
GABRA1-53kD

Figure S3

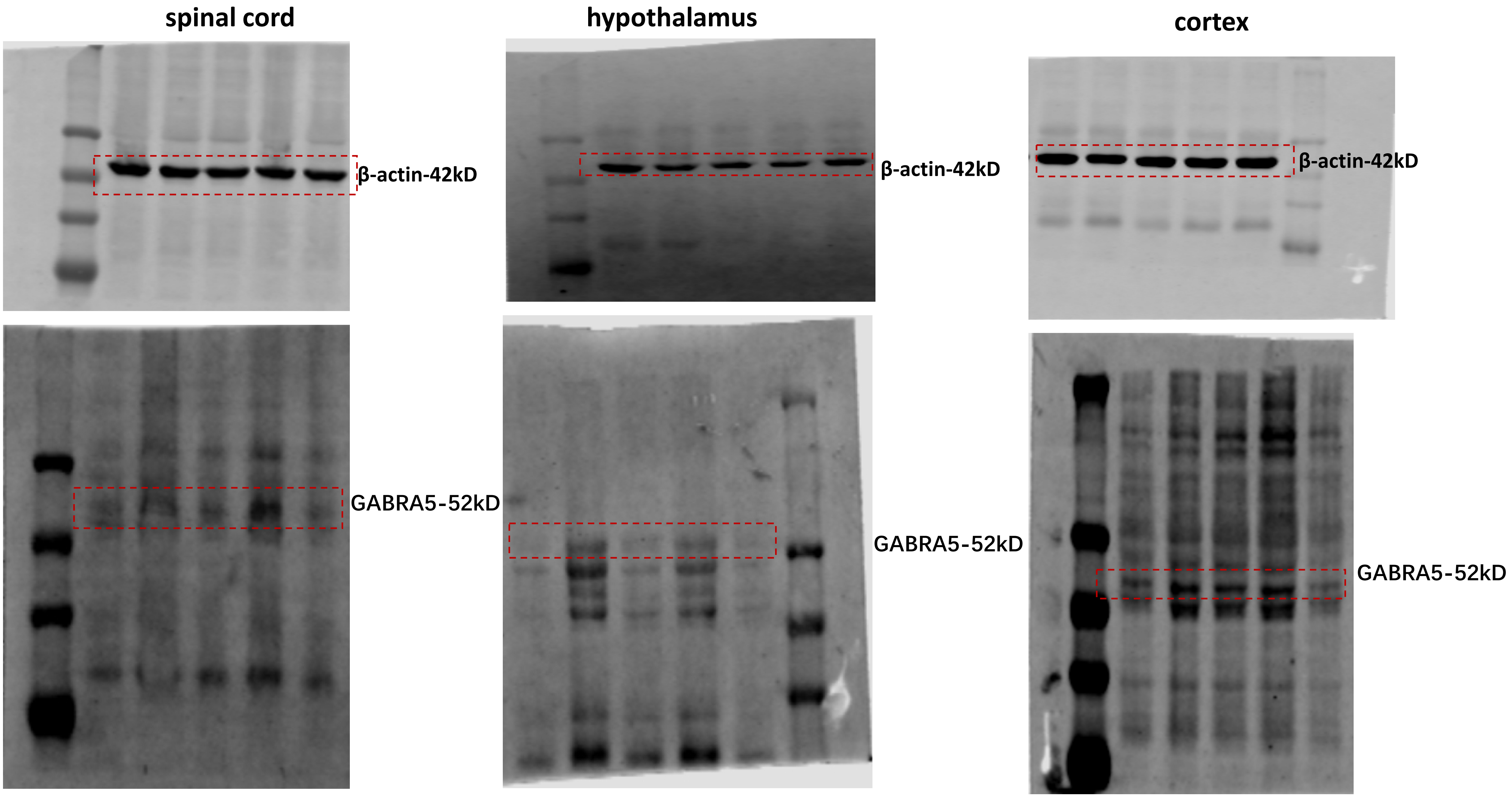
spinal cord
hypothalamus
cortex
β-actin-42kD
β-actin-42kD
β-actin-42kD
GABRA5-52kD
GABRA5-52kD
GABRA5-52kD
