# Supplementary material for: N‐Demethylsinomenine Relieves Neuropathic Pain in Male Mice Mainly via Regulating α2‐Subtype GABAA Receptors
Source: CNS Neurosci Ther. 2025 Jan 3;31(1):e70197. doi: 10.1111/cns.70197 (PMC11696256; doi:10.1111/cns.70197)
Supplement: Supplementary file 1 — Data S1. [file CNS-31-e70197-s001.zip › Supplementary Material-CNS-clean.docx]

**Supplementary Material**


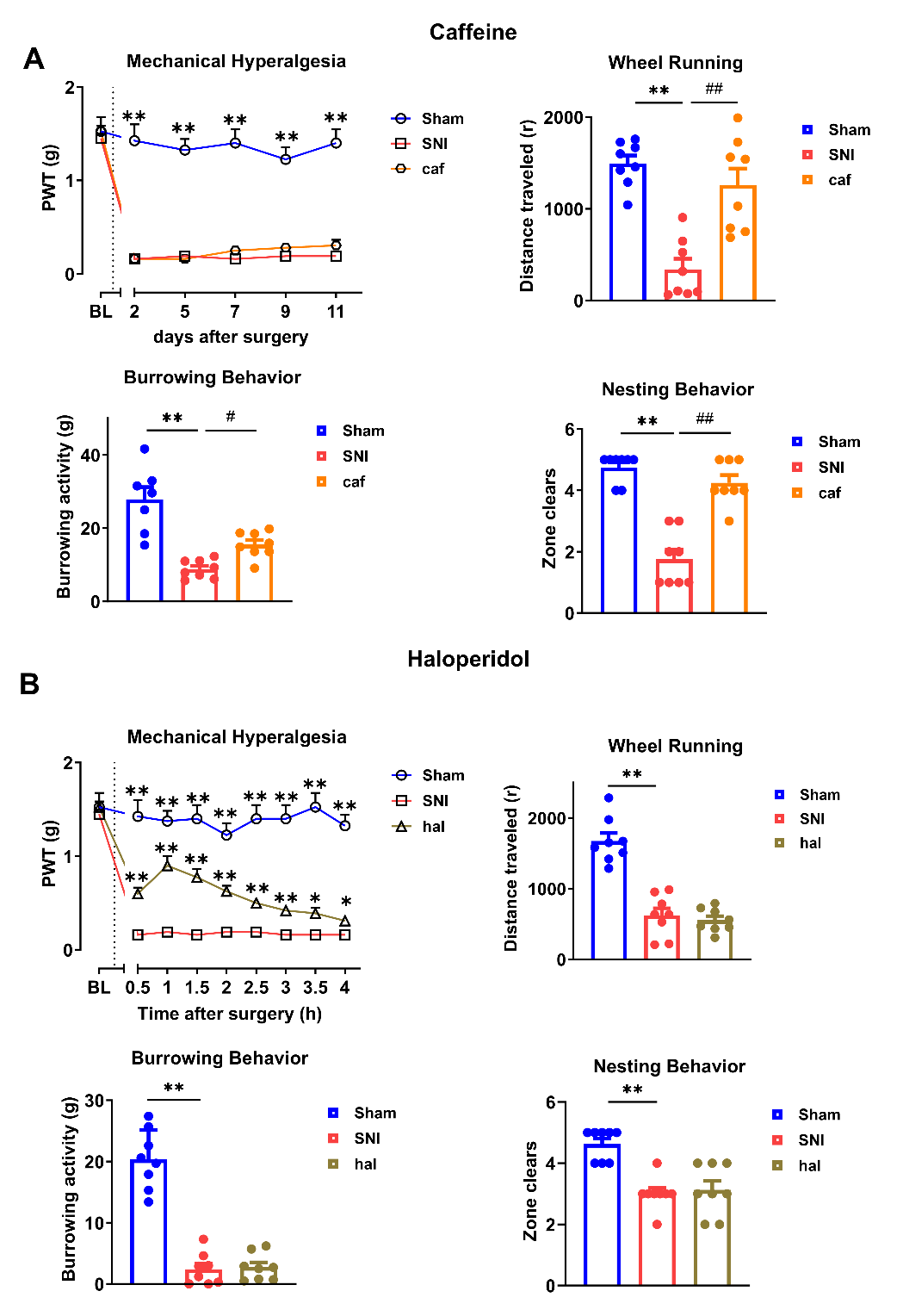


**Fig.S1** Analgesic effect of caffeine and haloperidol on a model of SNI induced neuropathic pain in mice. A: Effect of caffeine on the behavior of mice after SNI surgery; B: Effect of haloperidol on the behavior of mice after SNI surgery; BL represents the baseline value before SNI surgery, statistical analysis of line graph was performed using the two-way ANOVA analysis with repeated measures (Time × Treatment) followed by Bonferroni *post hoc* analysis (**p* < 0.05 and ***p* < 0.01 *vs.* the SNI group), and statistical analysis of bar graph was performed using the one-way ANOVA analysis followed by Bonferroni *post hoc* analysis (** p*< 0.05 and ***p* < 0.01 *vs.* the Sham group; #*p* < 0.05 and ##*p* < 0.01 *vs.* the SNI group), ANOVA F: caffeine, mechanical hyperalgesia (F(5, 126)=42.27, F(2, 126)=188.8), wheel running (F(2.000, 15.38)=21.41), burrowing behavior (F(2.000, 8.677)=20.13), nesting behavior F(2, 21)=41.33); haloperidol, mechanical hyperalgesia (F(4.916, 103.2)=54.98, F(2, 21)=50.35), wheel running (F(2, 21)=43.77), burrowing behavior (F(2, 21)=72.20), nesting behavior F(2, 21)=15.70)


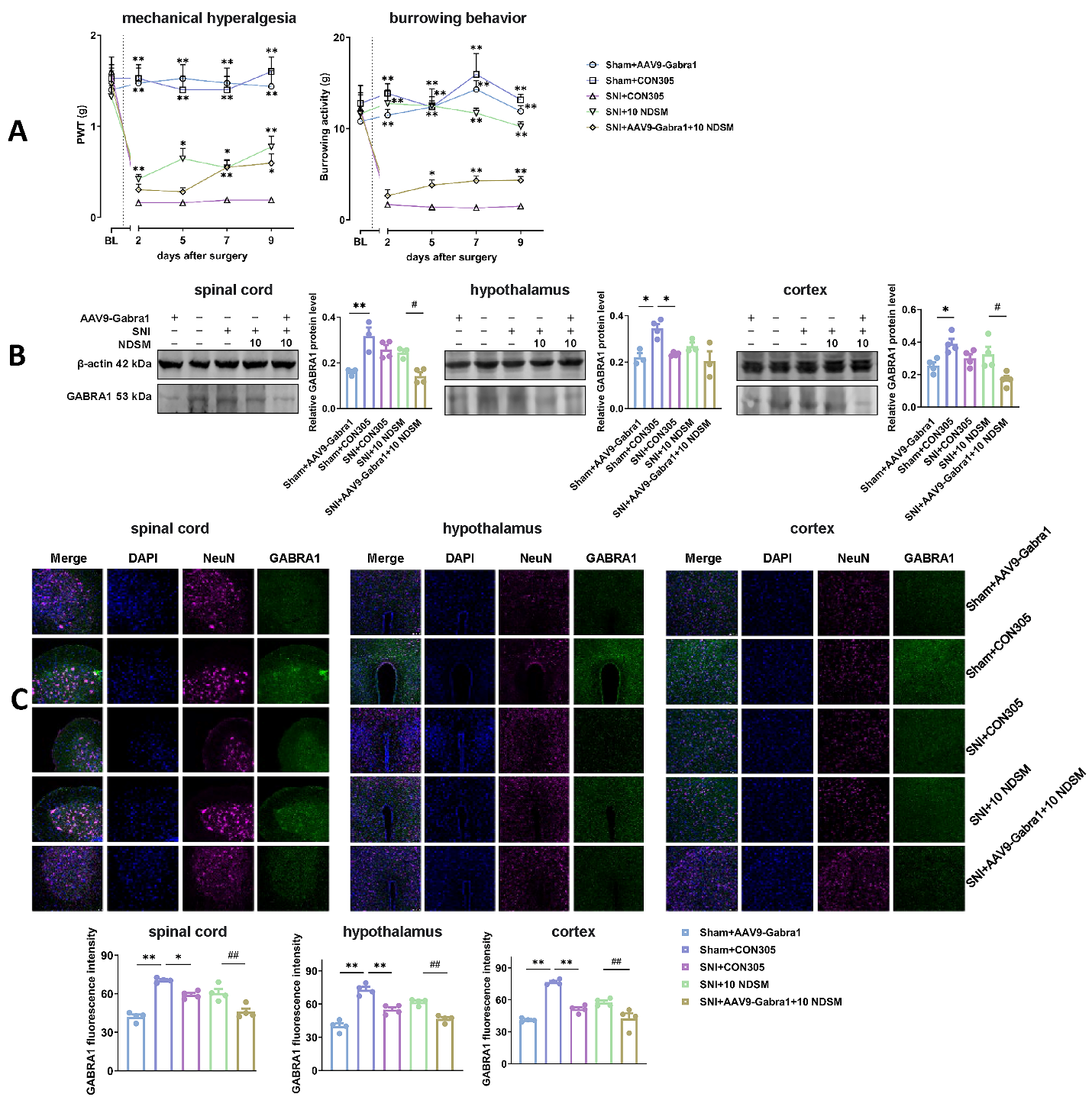


**Fig.S2** Gene silencing of GABRA1 in the spinal cord and brain regions on the analgesic effect of NDSM. A: Effect of intrathecal injection of AAV9-Gabra1-RNAi virus on PWT and burrowing activity in mice induced by SNI surgery. BL represents the baseline value before the SNI surgery. Statistical analysis was performed using the two-way ANOVA analysis with repeated measures (Time × Treatment) followed by Bonferroni *post hoc* analysis (**p* < 0.05 and ***p* < 0.01 vs. the SNI+CON305 group); B: WB assay of GABRA1 expression in the spinal cord, hypothalamus and cortex of mice; C: Immunofluorescence of GABRA1 in the spinal cord, hypothalamus, and cortex of mice; n = 4 (each set of data allows up to one outlier to be eliminated), CON305 is a negative control virus, statistical analysis of WB and immunofluorescence was performed using the one-way ANOVA analysis followed by Bonferroni *post hoc* analysis (**p* < 0.05 and ***p* < 0.01 vs. the SNI+CON305 group; #*p* < 0.05 and ##*p* < 0.01 vs. SNI+10 NDSM group). ANOVA F: mechanical hyperalgesia (F(2.712, 94.90)=36.43, F(4, 35)=35.92); burrowing behavior (F(2.788, 97.57)=9.213, F(4, 35)=81.55); WB (spinal cord, F(4, 12)=11.79, hypothalamus, F(4, 12)=7.144, cortex F(4, 15)=7.266); IF( spinal cord, F(4, 15)=29.68, hypothalamus, F(4, 14)=37.60, cortex F(4, 14)=32.57)


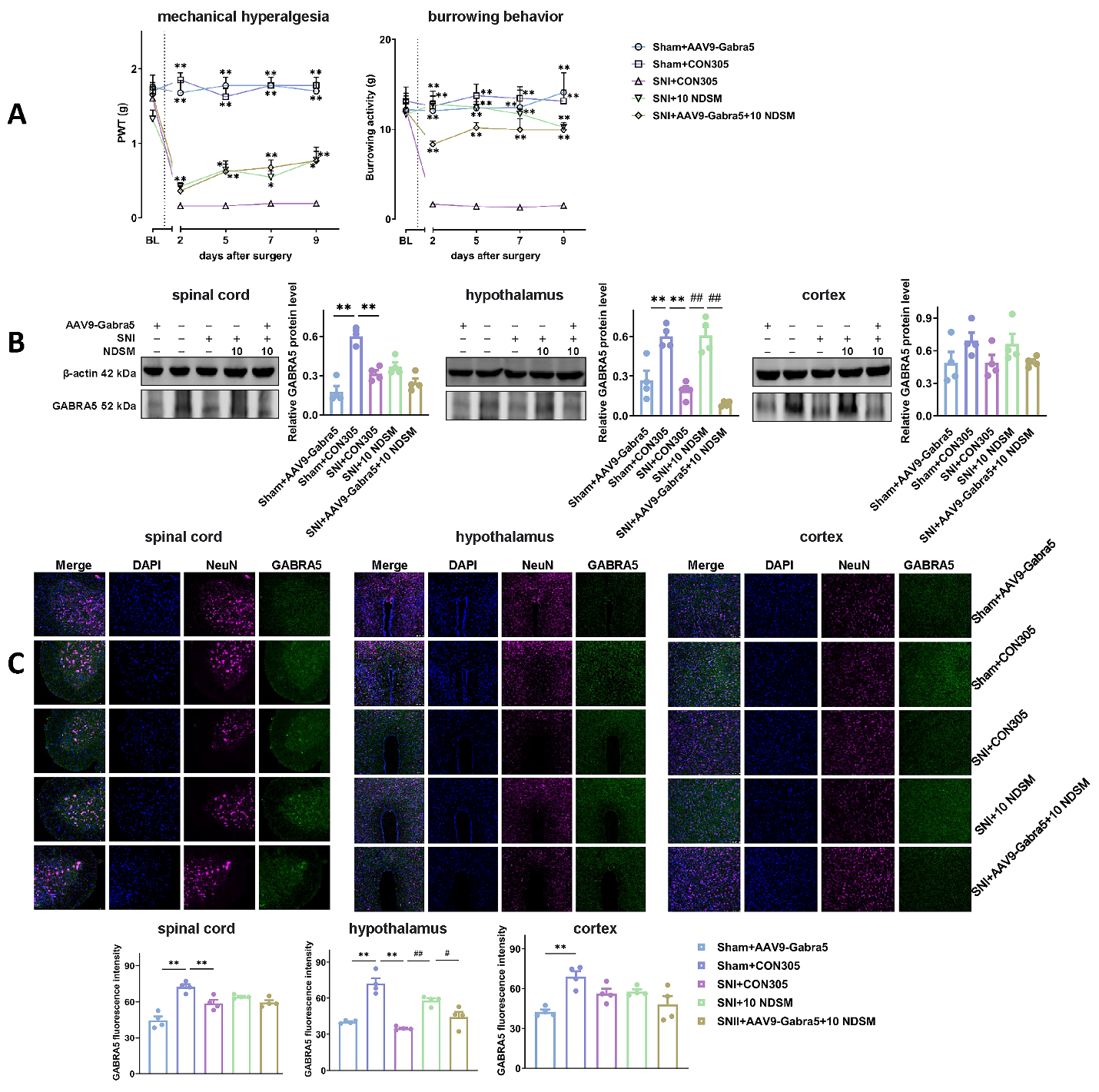


**Fig.S3** Gene silencing of GABRA5 in the spinal cord and brain regions on the analgesic effect of NDSM. A: Effect of intrathecal injection of AAV9-Gabra5-RNAi virus on PWT and burrowing activity in mice induced by SNI surgery. BL represents the baseline value before the SNI surgery. Statistical analysis was performed using the two-way ANOVA analysis with repeated measures (Time × Treatment) followed by Bonferroni *post hoc* analysis (**p* < 0.05 and ***p* < 0.01 vs. the SNI+CON305 group); B: WB assay of GABRA5 expression in the spinal cord, hypothalamus and cortex of mice; C: Immunofluorescence of GABRA5 in the spinal cord, hypothalamus, and cortex of mice; n = 4 (each set of data allows up to one outlier to be eliminated), CON305 is a negative control virus, statistical analysis of WB and immunofluorescence was performed using the one-way ANOVA analysis followed by Bonferroni *post hoc* analysis (**p* < 0.05 and ***p* < 0.01 vs. the SNI+CON305 group; #*p* < 0.05 and ##*p* < 0.01 vs. SNI+10 NDSM group) ANOVA F: mechanical hyperalgesia (F(2.965, 103.8)=50.91, F(4, 140)=63.85); burrowing behavior (F(4, 140)=5.129, F(4, 35)=33.10); WB (spinal cord, F(4, 14)=18.48, hypothalamus, F(4, 12)=7.144, cortex F(4, 15)=7.266); IF( spinal cord, F(4, 15)=18.83, hypothalamus, F(4, 15)=26.28, cortex F(4, 15)=6.967)
